# Microbial Lipases and Their Potential in the Production of Pharmaceutical Building Blocks

**DOI:** 10.3390/ijms23179933

**Published:** 2022-09-01

**Authors:** César A. Godoy, Juan S. Pardo-Tamayo, Oveimar Barbosa

**Affiliations:** 1 Laboratorio de Investigación en Biocatálisis y Biotransformaciones (LIBB), Grupo de Investigación en Ingeniería de los Procesos Agroalimentarios y Biotecnológicos (GIPAB), Departamento de Química, Universidad del Valle, Cali 76001, Colombia; 2 Grupo de Investigación de Materiales Porosos (GIMPOAT), Departamento de Química, Universidad del Tolima, Ibague 730001, Colombia

**Keywords:** microbial lipase, building blocks, Active Pharmaceutical Ingredients (APIs), lipase catalytic properties, promiscuity, lipase immobilization

## Abstract

Processes involving lipases in obtaining active pharmaceutical ingredients (APIs) are crucial to increase the sustainability of the industry. Despite their lower production cost, microbial lipases are striking for their versatile catalyzing reactions beyond their physiological role. In the context of taking advantage of microbial lipases in reactions for the synthesis of API building blocks, this review focuses on: (i) the structural origins of the catalytic properties of microbial lipases, including the results of techniques such as single particle monitoring (SPT) and the description of its selectivity beyond the Kazlauskas rule as the “Mirror-Image Packing” or the “Key Region(s) rule influencing enantioselectivity” (KRIE); (ii) immobilization methods given the conferred operative advantages in industrial applications and their modulating capacity of lipase properties; and (iii) a comprehensive description of microbial lipases use as a conventional or promiscuous catalyst in key reactions in the organic synthesis (Knoevenagel condensation, Morita–Baylis–Hillman (MBH) reactions, Markovnikov additions, Baeyer–Villiger oxidation, racemization, among others). Finally, this review will also focus on a research perspective necessary to increase microbial lipases application development towards a greener industry.

## 1. Introduction

Lipases or triacylglycerol acyl hydrolases (EC 3.1.1.3) are ubiquitous in all realms of life. In nature, they are mainly characterized by catalyzing the hydrolysis of triglycerides and long-chain partial glycerides, releasing fatty acids, monoglycerides and glycerol [1,2]. These enzymes constitute a key link in the cellular processes related to the absorption, release and metabolism of fats [3,4,5,6,7], since its substrates and derivatives are the most abundant lipids in cells. On the other hand, some lipases have also been attributed functions as defense enzymes [7,8] or as virulence factors [9]. 

Lipases are the most used enzymes in biotechnological applications worldwide; together with carbohydrases, nucleases and polymerases, their market was estimated at 425 million USD in 2018 with a Compound Annual Growth Rate (CAGR) of 6–8% [10,11]. Their applications cover different areas such as clinical diagnosis, bioremediation, the production of detergents, food, polymers, biofuels and fine chemicals, from which a large number of patents are derived [1,2,10,12]. The preference for the use of lipases in industrial applications over enzymes belonging to other classes lies, at least in part, in that lipases do not need costly or difficult to regenerate cofactors such as dehydrogenases or ligases [1].

A particular advantage of lipases of microbial origin compared to those of other origins is that no colipases are required for activity, as is the case with some pancreatic lipases found in mammals [13]. In addition to the above, microbial lipases have a greater range of operating conditions (pH, temperature, ionic strength, a_w_, etc.) as a consequence of the adaptation of its produced microorganisms to the environmental conditions, which in some cases can be extreme [14]. Furthermore, microbial lipases are typically active as monomers, relatively small (19–65 kDa) and have at least 170 structures resolved to date (rcsb.org): this makes it easier to elucidate their mechanisms of action and design variants in a more rational way by using tools of molecular biology such as directed evolution, among others [15,16,17,18]. This also has favored their heterologous production in easily cultured organisms such as fungi and bacteria, allowing their large-scale production [10,11,16].

The attractiveness of microbial lipases in applications also lies in their versatility, by facilitating the transfer of acyl groups from a wide number of substrates, not only to water (its natural substrate), but also to various nucleophiles such as H_2_O_2_ [19], alcohols (including derivatives like sugars) [20]), amines [21], amino acids [22], amino alcohols [23] and thiols [24], among others, which results in a wide potential for application in organic synthesis reactions as those involved in the production of building blocks of Active Pharmaceutical Ingredients (APIs). To thermodynamically favor synthesis over hydrolysis reactions, it is necessary that the reaction media maintain, among other characteristics, a low aqueous activity (a_w_). This involves using organic solvents or non-conventional media such as ionic liquids or supercritical fluids [25,26]. Interestingly, in this type of media and in the presence of suitable substrates, it has been observed that some microbial lipases may present promiscuity, expanding their range of applications to useful reactions in organic syntheses such as the Michael addition [27], aldol condensation [28], Mannich [29], Baeyer–Villiger oxidation [19], Knoevenagel [30], Morita–Baylis–Hillman Adduct Formations [31], Kabachnik–Fields [32], and racemization [33], among others.

Regardless of the type of reaction catalyzed by microbial lipases, their ability to transfer groups in a (regio-, chemo-, stereo-) selective way gives these enzymes the ability to generate products with high optical purities [34,35]. This is quite attractive since the reactions oriented towards the production of chiral building blocks generally involve chemosynthetic processes that, even today, present the greatest challenges when it comes to complying with the principles of green chemistry. For example, the E-factor (the ratio of the mass of waste per mass of product) that characterizes them is the highest within the different industries related to chemical transformations, reaching values of 100 or higher [36]. Thus, the implementation of lipases for the intensification of these processes, given their advantages as a biocatalysts, can provide a positive contribution to increase the sustainability of the production of APIs and related building blocks [35,37].

However, not all are advantages: the stability, selectivity and activity of lipases (and other enzymes) can be affected when the reaction conditions differ from those that occur under natural conditions; the presence of concentrated synthetic substrates, organic solvents, or additives and in general, when the values of the thermodynamic parameters such as pH, T, P and concentration, differ from those in vivo [26,38,39]. Furthermore, one of the main hurdles to increasing the use of lipases in the industry is their high (economic) price, which, if compared with conventional catalysts, can be up to five hundred times or more. (e.g., price per gram of *Candida antarctica lipase* B (CAL B) vs. NaOH (sigmaaldrich.com, accessed on 4 March 2020). 

Therefore, in order to make the use of lipases economically viable in the industry, it is necessary to facilitate their reuse in several production cycles while maintaining adequate catalytic properties: at the end of these cycles, the ratio per unit of time between the mass of product obtained/mass of spent catalyst must be comparable to that of the catalyst or conventional process to be substituted [35,40]. The reuse of a lipase supposes facilitating its physical separation from the reaction medium, for which various immobilization strategies are available, including: entrapment, co-crystallization, lyophilization, cross-linking, mineralization, physi- and/or chemo- sorption on solid surfaces, etc. [41,42,43,44,45]. Particularly, one of the strategies most used in the industry is the adsorption of enzymes on solid surfaces that are part of meso- and macroporous supports; mainly due to its high commercial availability, variety and relative low cost, which facilitates their scaling [44,46]. Another advantage is that in this type of support, strategies have been applied successfully to recover microbial lipases that have been inactivated by the effect of organic solvents or reaction conditions [40,47,48,49,50,51].

This review encompasses mostly the last seven years of the most prominent applications of lipases, including those immobilized on porous supports as a complement or alternative per se greener compared to the usual strategies of organic synthesis, related to the production of APIs. In this sense, initially a general description will be made of the structure–function relationships of microbial lipases, applications of these enzymes in reactions of interest (Novozym^®^ 435 and others), considerations when immobilizing lipases to modulate their properties in applications and lipases and their derivatives as promiscuous catalysts to finally propose research paths in order to favor the applications of lipases in the synthesis of building blocks of APIs.

## 2. Microbial Lipases: Structure and Function

In this section, the main aspects of the structure–function of microbial lipases, reviewed more extensively in other articles, will be summarized in a general way [52,53,54,55], but, here, emphasis will be placed on aspects to be discussed in later sections such as their immobilization and applications in reactions of interest in the production of compounds related to APIs.

### 2.1. Structure

**Figure 1:**
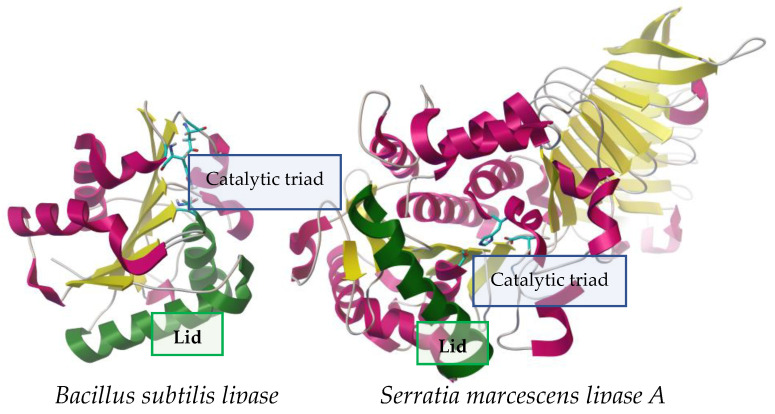
Contrast between two lipases of different sizes from Bacillus subtilis (19 kDa, left) [58] and Serratia marcescens (64.9 kDa, right) [59], showing structural similarities such as: lid (represented in green), active center (represented by sticks), α-helices (pink) and β-lamina (yellow). PDB structures 5cri (Bacillus subtilis) and 2aqua (Serratia marcescens), visualized with AutoDockTools 4.2.

In nature, lipases (EC 3.1.1.3) or triacylglycerol acyl hydrolases mainly catalyze the total or partial hydrolysis of ester bonds in triglycerides, usually in the aggregate state and at the level of such surfaces, however, other substrates on which they can act in vivo are not ruled out, such as cholesterol esters, phospholipids, lysophospholipids and ceramides, as has been observed for some lipases [56,57]. Microbial lipases are generally globular enzymes with different sizes: an example of a low molecular weight lipase is that of Bacillus subtilis with 19 kDa [58], while at the other extreme is lipase A from Serratia marcescens with 64.9 kDa [59] (Figure 1). These enzymes may present structural metal cations such as Ca^2+^ or Zn^2+^; the first related to activity and stability, while the case Zn^2+^, with thermostability and a tendency to generate aggregates occurs with the lipases of Geobacillus stearothermophilus or Geobacillus thermocatenulathus [54,60,61].

Regardless of their origin, lipases are characterized as serine hydrolases in which the catalytic Ser is found within a consensus sequence (Gly-X1-Ser-X2-Gly); this sequence may have variants that, together with the biological origin of the enzyme, will define its classification within the eight proposed families [62]. The catalytic Ser is part of a set of three amino acids together with His and Asp (or Glu) residues, called the catalytic triad. In the primary structure, this Ser is located closer to the N-terminus, while His is generally located closer to the C-terminus [55,63,64,65,66,67]. In terms of the secondary structure, the consensus sequence together with the catalytic triad are contained within the α/β hydrolase fold: it consists of a central beta sheet composed of eight parallel beta strands (1 to 8) connected by up to six alpha helices (A–F). In general, in this fold, the Ser is located at the C-terminal end of the β5 strand (within the consensus pentapeptide) characterized by the beta-turn-alpha(αC) motif referred to as the “nucleophilic elbow”. Near this elbow are found the other components of the triad: Asp (or Glu) between αE and β8, which is part of a long independent loop, while His is usually located between αF and β8 as part of a long loop [63,64,65,66,67]. The catalytic Ser and amido groups C_α_ (e.g.*,* from Gln and Thr) and OH (from Thr or Tyr) will conform the “oxyanion hole”; this assembly in turn constitutes the deepest end of the predominantly hydrophobic cavity where the oxyanion and the acyl acceptor will join [68,69]. 

Sometimes the catalytic cavity will be more or less exposed to the solvent depending on the presence and state of a self-regulatory mobile domain called the lid (or flap) [52]. The lid has an amphipathic and flexible nature and is composed of one or more α-helices and different loops [52,63,64,65,66,67]. According to evidence from X-ray structure and molecular dynamics, in the case of lipase from Pseudomonas sp. MIS38, the ability to move this lid would be related to the presence of structural metal ions (Ca^2+^ or Zn^2+^) that function as hinges or attachment points of the lid depending on the lipase [66,70]. Thus, the lipases that contain lid will present a conformational equilibrium between an open or closed form, depending on the state in which it is found. This has been supported by the large number of lipases such as those from Thermomyces lanuginosus (TLL), Candida (or Pseudozyma) antarctica (CAL B), Geobacillus thermocatenulatus (BTL2), among others, which have been crystallized in different conformations and where it has been shown displacements and reorganizations in the secondary structure of the lid between one state and another: open conformations have not only been observed in lipases co-crystallized with substrates, detergents or inhibitors, but also when they are forming dimers, in which case the lids would mutually constitute part of the binding interface [63,64,65,66,67,71]. In addition to this evidence, there are other studies with lipases in solution, which through techniques such as: Single Particle Tracking (SPT, Section 2.2.2), Site-Directed Spin Labeling (SDSL), Site-Directed Fluorescence Labeling (SDFL) and Electron Paramagnetic Resonance Spectroscopy (EPR), have shown that lid-containing lipases present different conformational states depending on the presence of substrates, detergents and, in general, substances that, upon aggregation, assemble hydrophobic interfaces with water [72,73,74]; this behavior has also been evidenced in silico through molecular dynamics studies [75,76,77].

### 2.2. Mechanism of Action: Interfacial Activation and Catalysis

#### 2.2.1. Interfacial Activation

As is known, the natural substrates of lipases are mainly triglycerides of fatty acids (TG) characterized by their low solubility in water where they tend to form aggregates. On the other hand, microbial lipases are usually produced as globular enzymes that tend to be soluble in the cellular or extracellular environment of an aqueous nature. This means that, evolutionarily, lipases must have acquired characteristics that allowed them to access these insoluble substrates from the aqueous medium, which necessarily involves a transition to a more hydrophobic conformation through structural changes that do not result in their inactivation or denaturation, but instead lead to another more active folded state: this explains, in part, why some lipases are stable even in the presence of non-aqueous solvents unlike other enzymes such as aldolases (See Molecular Origins of the Catalytic Promiscuity of Lipases) [78].

The above implies that in aqueous media or in the absence of substrates (and their analogs), the majority population of lipase molecules (with lid or flap) shows a mainly closed conformation where the lid interacts through hydrophobic side chains with the outermost part of the catalytic cavity [52,66]. In contrast, in the presence of aggregated substrates, lipase experiences an increase in the proportion of its hydrophobic area characterized by a new conformation that constitutes the dominant population of lipase [52,72,75,79].

**Figure 2:**
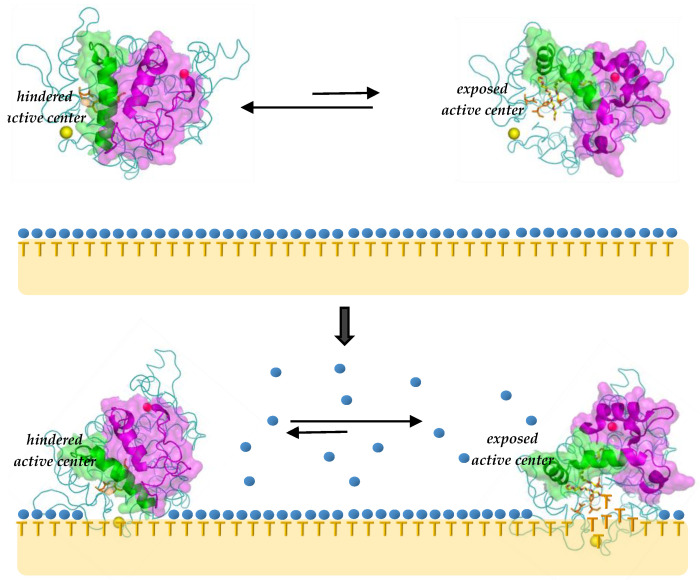
Scheme of open-closed conformational equilibrium of lipases (i.e.*,* BTL2, PDB 2w22). Upper panel (equilibrium in water): closed form (**upper left**) and the less populated open form (**upper right**). Yellow and purple spheres represents lipase structural ions. Blue spheres represent clathrate water molecules and “T” for triglycerides, both at the interphase. Lower panel: after lipase adsorption, some clathrate water molecules get desorbed and disordered as bulk water. Some individual “T” (substrate) molecules can now diffuse towards the active center of the more stable open lipase form (**lower right**), starting catalysis [80].

The presence of a TG-water interface and an adequate orientation of the lipase [80] towards the surface of TGs would shift the conformational equilibrium to a form in which the lid domain constitutes part of the interaction surface with the substrate surface added, leaving the catalytic cavity accessible, resulting in an increase in activity. The main thermodynamic engine of the phenomenon described is the so-called “hydrophobic effect” (Figure 2) [81]: the displacement of clathrate water molecules from the hydrophobic surface of the adsorbent (here the one made up of aggregates of TG molecules) generated by the adsorbate (in this case, the enzyme) produces an increase in the entropy of the system. This not only generates an increase in the detected catalytic activity or “interphase activation” but may also imply the stabilization of the adsorbed lipase as a consequence of the reduction in the conformational space available for the unfolded state [82]: this type of phenomenon of activation/stabilization would also occur in the case of lipases immobilized on macroporous solid supports of a hydrophobic nature, as will be seen later (Section 3.1).

#### 2.2.2. Catalysis of Lipases Functioning as Serine Hydrolases

Once the lipase is adsorbed and its open conformation is stabilized, the transport of substrate molecules from the interface to its catalytic cavity will be favored; this has been evidenced by molecular dynamics studies where the M37 lipase in the open state acquires an angled position in front of the hydrophobic aggregated substrate surface and from there a molecule of the substrate is “pulled” until it is located in the active center, finally forming the Michaelis complex [77]. Although it is not explicitly indicated why the substrate molecule is “pulled”, it is understood that it is positioned in such a way that its reactive groups, of a more polar nature, are attracted towards the area of the catalytic cavity more polar, in other words, where the catalytic Ser and the oxyanion hole are.

**Figure 3:**
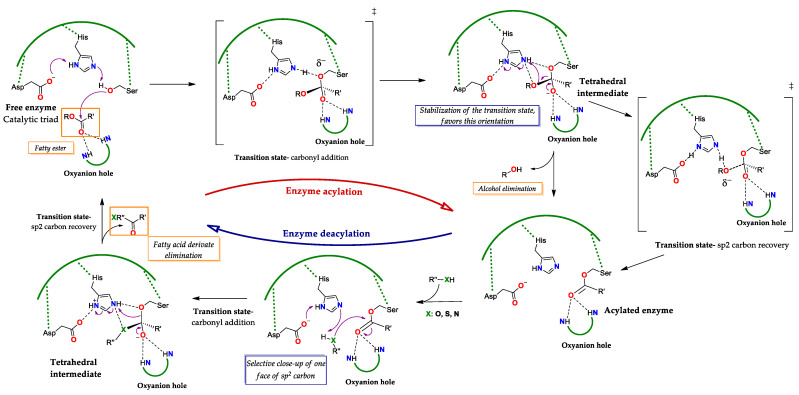
General catalytic cycle of lipases. The reaction starts by the nucleophilic attack of the catalytic Ser to the substrate, passing through a transition state (‡) of addition to the carbonyl to the generation of the selective tetrahedral intermediate (here, the R stereoisomer) where the negative charge density is concentrated in the O(β) of the Ser, here the preference towards the stereoisomer R is explained by the fact that the most stable transition state (‡) is the one where this orientation is favored. Next, the sp^2^ carbon is recovered, through a transition state (‡) here the charge density is focused on the O of the alkoxy group to be eliminated, in order to obtain the acylated enzyme. Subsequently, in the presence of a nucleophilic molecule type RXH, where X can be O, S or N, a tetrahedral intermediate is regenerated, by the addition transition state (‡) to one face of the sp^2^ carbon of the carbonyl. Finally, the sp^2^ carbon is recovered, producing a fatty acid derivative and regenerating the free active site. Adapted from [18,83,84].

Regarding the Michaelis complex, QM/MM studies have shown that the formation of E-S_1_ occurs with the “acyl donor” substrate and involves polar interactions between its O carbonyl with three H bonds donor groups in the oxyanion hole: two –NH residues from amino acids of the peptide chain, such as Gln and Thr, and one with the β-OH group of the side chain of the latter. As a consequence, the C carbonyl remains close to the O(β)H group of the catalytic Ser (Figure 3): It is important to point out that the interactions between the oxyanion hole as H bond donor and as carbonyl O acceptor will be maintained in the different transition states and intermediates [18,83,84]. These studies have shown that lipases, such as CAL B (or PAL B), employ a concerted mechanism; here, the transition state of reaction of the O(β) with the C carbonyl of S_1_ proceeds as H forms a bond of hydrogen with the imidazole N(ε) of the His of the triad, which supposes the shortening of the bond between the N(δ)H and the carboxylate (γ) of the Asp of the triad [18]. As a result, the transition state the C carbonyl of S1 becomes polarized, adopting a sp^3^ configuration and the O carbonyl acquires a negative charge. Thus, the tetrahedral intermediate results from the formation of the covalent bond between the O(β) of Ser with the now sp^3^ carbon (formerly sp^2^ carbonyl) of the substrate, the acquisition of negative charge of the carbonyl O (oxyanion) and the protonation of the N(ε) of His and of the carboxylate γ of Asp within the triad (Figure 3). This intermediate undergoes a charge rearrangement that involves the formation of a second transition state, which leads to the exit of the alkoxy residue from the substrate to which H is transferred from the N(ε) group of His, and, in the formation of an ester-type bond between Ser and the acyl moiety of the substrate, an intermediate known as “acylated enzyme”. The arrival of the acyl acceptor substrate “S_2_” of a nucleophilic nature (normally water), implies the formation of a third transition state, in which the S_2_ molecule forms interactions as a donor of an H bond with the basic N(ε) of His and as a donor of an electron pair of its O atom with the carbonyl C of S_1_ (in the acylated enzyme), causing the loss of its sp^2^ character (Figure 3). This last interaction leads to the formation of a covalent bond between the O of S_2_ with the C carbonyl of S_1_, forming a second tetrahedral oxyanion-type intermediate and the protonation of N(ε). The weakening of the bond between the O(β) of Ser and the C carbonyl of S_1_ occurs at the same time as an interaction with the H of N(ε) and a transition from sp^3^ to sp^2^ in the C carbonyl is established, which generates the last transition state. Finally, the formation of the acylated product is achieved by breaking the O(β) bond with the C carbonyl while the protonation of the catalytic Ser occurs, reestablishing the initial state of the enzymatic triad and the release of the product (Figure 3). These QM/MM studies are consistent with the Ping-Pong Bi-Bi mechanism used to kinetically model reactions catalyzed by free or even adsorbed (immobilized) lipases on hydrophobic substrate surfaces (Section 2.2.1), reactions including not only hydrolysis but also alcoholysis, acidolysis and transesterification [85].

Interestingly, something not well studied is that the consequences of the catalysis phenomenon described above, which focuses within the catalytic cavity, also seems to have a reflection outside of it: using the Single Particle Tracking (SPT) assay, it was determined that in the extent that lipases (e.g.*,* TLL) catalyze the hydrolysis of aggregated triglycerides, the products of the reaction (alcohols and acids fatty) would dynamically affect the state of the adsorbed lipase: these products would function as “lipase repellent coatings”, so that the enzyme diffuses towards more hydrophobic surfaces, a kind of “chemotaxis” guiding the lipase to surfaces where the concentration of the unprocessed substrate is higher, thus favoring a higher catalytic efficiency [74]. These types of studies open an opportunity to study thermodynamic and kinetic aspects less explored in the catalysis of lipases, such as, for example, how the composition of the interface on which the enzyme is adsorbed, or lid modifications, affect the activity or selectivity of the lipase. This would have applications in the production of APIs that facilitate their production in the presence of interfaces, for example, as occurs in the kinetic resolution of esters containing azaspirononanes or spiro-β-lactams by the lipase of *Arthrobacter* sp. [86], respectively. These compounds were resolved in a biphasic system of diisopyopyl ether/phosphate buffer and are related to homoharringtonine, a molecule approved for the treatment of chronic myeloid leukemia and in the production of antibiotics [86,87]. 

#### 2.2.3. Selectivity of Lipases

Lipases can catalyze reactions involving natural lipids that, in addition to TGs, can include the group of phospholipids: some of these are bioactive (phosphatidic acid or lysophosphatidylcholine) and others, such as lecithins, are used in the pharmaceutical industry as emulsifiers or for the release of active principles when they are part of liposomes, such as those present in the first nanodrug approved by the FDA, the anticancer Doxil^®^ [88,89]. Given their structural similarity, these phospholipids can be obtained naturally or synthetically from TGs as sources of fatty acids, some of which are of interest in human health, such as PUFAs (polyunsaturated fatty acids) [90]. Other derivatives have also shown potential as prodrugs to mitigate the side effects of mefenamic acid, an anti-inflammatory [91]. Due to the great variety of natural TGs and their abundance in biomass, it has been important to establish connections between their structure and the observations obtained during their enzymatic transformation in order to obtain derivatives of commercial interest. This has enabled a greater understanding of the structural origins of the selectivity of lipases and extrapolate some of their implications on synthetic substrates, as shown below:

#### Chain Length

It has been observed how the lid and its dynamics influence substrate selectivity in lipases [52], also conditioning the adsorption of TLL on the hydrophobic surface of substrates (orientation, activation and stability) and its diffusion on it [74,76]. It is important to note that not only the catalytic cavity but also the lid can be part of such substrate binding surfaces. The structural model of Pleiss et al. have postulated that the size, polarity, and shape of the lipase catalytic cavity (crevice, funnel, or tunnel) will determine the regio- and enantioselectivity of the lipase [69]. Through structural comparisons between a group of eight esterases and lipases, they showed that the catalytic cavity of the latter is usually larger, essentially to accommodate comparatively more hydrophobic and larger substrates such as medium and long chain TG. For example, in esterification or alcoholysis reactions, the fact that CAL B (or PAL B) had a cleavage site for the acyl residue (scissile fatty acid binding site) at the height of the carbon chain up to the C13 carbon atom while that the Rhizomucor miehei lipase (RML) presented it up to C18 explained the preference of the latter for longer chain substrates [69].

Mutant lipases that present blockades in scissile sites of the acyl residue or that are more hydrophilic tend to decrease their preference for long-chain TGs (triolein in relation to short-chain substrates (tributyrin)) in lipases from Rhyzopus sp. [92]; something similar was observed with variants (Gly237Ala/Leu/Val/Tyr) of CAL A, modified by directed mutagenesis that generate blockages at the entrance of a binding tunnel where part of the long chains of the substrate were accommodated in the native enzyme [93]. Lately [94], for this enzyme, the 217–245 helix-loop-helix motif was identified as a key in TG recognition: mutants with a single substitution that conferred discrimination between short versus long TGs, for this enzyme, the 217–245 helix-loop-helix motif was identified as a key in TG recognition: mutants with a single substitution that conferred discrimination between short versus long TGs were identified at positions 183, 235, 240, 244, 338 and 377, while those with a preference for long TGs had substitutions at positions 84, 93, 232 and 359.

#### Cis/Trans Selectivity

Structural evidence of this type of selectivity has been obtained by studying lipases of known structure in esterification reactions against geometric isomers of 9-octadecenoic acid in cis (oleic) and trans (elaidic) and with cis/trans and positional isomers of conjugated linoleic acid (CLA) [95,96]: in general, and following the analyzes of Pleiss et al., enzymes with wider binding cavities are better at catalyzing reactions of the cis vs. trans isomer of 9-octadecenoic acid as RML, while those with deeper and narrower cavities (fit better with the trans isomer) such as CAL A show a higher trans/cis selectivity compared to the previous enzyme; in the case of the CLA isomers, no clear trends were observed, since, due to the conformations that such substrates could acquire, not many differences are expected in terms of their shape. In other study [97], it was observed that the Phe149, Tyr221, Phe222, Ile301, and Leu305 residues were crucial in the generation of binding tunnels to straight or curved acyl residues, favoring selectivity for trans (or saturated) or cis fatty acids, respectively.

#### Preferences between tri, di- and Mono Glycerides

Within the lipases, there are also differences in the chemoselectivity of the type of glyceride they prefer to react with. More selective lipases against partial glycerides (mono- and diacylglycerol lipases, MDGLs) have a wide interest in applications in the food, nutraceutical and biofuel industries [98,99]. In Malassezia globosa lipase (SMG1), the selectivity against mono- and di-glycerides would result from the presence of two bulky and hydrophobic residues (Trp, Phe) adjacent to the catalytic site and a long N-terminal hinge region of the lid that can interact as a steric hindrance for the entry of TGs [100]. A mutant Q282L of SMG1 revealed that Gln in the native enzyme, being less hydrophobic than Leu in the mutated one, would be partly responsible for a less favorable interaction with TGs, explaining the null activity of the native enzyme against the latter [101]. 

**Figure 4:**
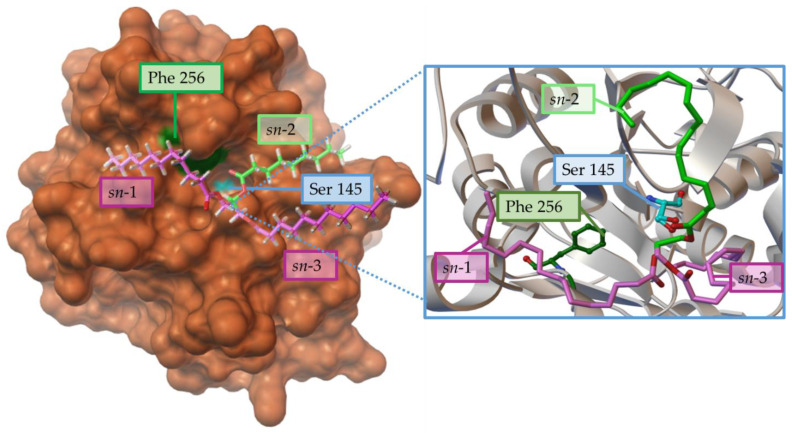
Steric effects of the residue Phe256 (Green) from Penicillium cyclopium lipase prevent the adequate interaction of sn-1 position (violet) in the substrate triacylglycerol (cyan) with the catalytic triad represented in orange, but this effect about the carboxyl carbon from the sn-2 position to the O(β) of Ser145. Structure from PDB 5ch8, using AutoDockTools 4.0 viewer and adapted from [102].

Taking as reference the Aspergillus oryzae MDGL-like lipase (AOL), also with bulky residues (Trp, Phe), at the entrance of the catalytic cavity, a consensus sequence Phe-X_1_-X_2_-His was identified for the MDGLs of the RML family. This sequence would form a “bridge-like” structure that would govern the recognition of substrates by preventing the accommodation of the sn-2 chain in the case of a TG [102]. Something similar has been observed in the recombinant lipase from Penicillium cyclopium (rePcMdl), where the Phe256 residue would prevent an adequate interaction from the sn-1 chain of the TG (Figure 4). 

#### Regioselectivity

As indicated, in nature TGs are the natural substrates of lipases. Due to their structure they have three positions (stereospecific numbering system) through which they can be hydrolyzed, defined as sn-1, sn-2 and sn-3. Thus, lipases have been classified by their ability to hydrolyze positions 1 and 3 that are equivalent (sn-1,3 selective), position 2 (sn-2 selective) or nonselective. Lipases able to introduce palmitic acid in sn-2 position are of interest in the infant food industry, given their ability to produce from vegetable sources triglycerides similar to those found in human milk [103]. The sn-1,3 selectivity shown by lipases as those from Rhizopus oryzae (Lipase F AP-15^®^) in hydrolysis or in acylations, has enabled their use in applications such as in the production of phospholipids rich in DHA (docosahexaenoic acid) or in nutraceuticals with potential benefits for human health [88,104].

In studies with CAL B using kinetic measurements and molecular dynamics studies, an attempt was made to explain the influence of the solvent on its ability to esterify sn-1 or sn-2 positions of glycerol with oleic acid. It was observed that the sn-2 position tended to be favored proportionally to the log P of the solvent. This was due to an increase in electronic and Van der Waals interactions between the enzyme and the sn-2 position and a weakening with the sn-1 one, although, in absolute terms, the latter remained in the most stable position [105].

According to the mechanism described for serine hydrolases in the case of an esterification with glycerol, the enzyme is expected to form a covalent intermediate where the acyl group is relatively fixed by its interactions with the oxyanion hole and the catalytic Ser, while glycerol, in accordance with its orientation in the pocket where it resides, presents interactions with the nearby environment, especially an H bond as a donor with N(ε) of His that can be given from its –OH groups in position 1 or 2. Taking into account the above, the reason why the sn-1,3-selective lipases are more numerous (RML, TLL, CAL B, etc.) than the sn-2 or non-selective ones such as CAL A or that of Geotrichum candidum [103,106] could be that the positions that have primary OH groups in glycerol are more abundant, reactive and less hindered (assuming similar acyl lengths), so that acquiring an adequate orientation towards N(ε) of His would be more likely, favoring the sn-1,3 selectivity [107]. 

Regarding this type of positional selectivity, it must be taken into account that it can be affected by collateral reactions such as acyl migration present in partial glycerides. These migrations can occur spontaneously (especially at high temperatures) or be catalyzed by lipases at room temperature; the latter has been shown in density functional theory (DFT) studies [108]. An increase in acyl migration can also occur due to reaction conditions such as the use of polar solvents, high a_w_ or silica-type immobilization supports, etc. [109,110]. These aspects must be taken into account in API synthesis processes (or their building blocks) that generally contain adjacent –OH groups, as in the case of the presence of sugars and their derivatives [111,112] and in other polyols: an example is the case of derivatives of Flurbiprofenate, a powerful nonsteroidal anti-inflammatory drug (NSAIDs), which has been esterified with L-ascorbic acid (a polyhydroxy acid) through the use of different lipases (CRL, PPL and Novozym^®^ 435) in order to improve its absorption in the brain [113]. 

**Scheme 1:**
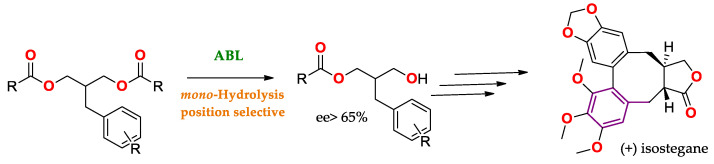
Mono-hydrolysis using ABL for the production of a precursor of (+) isostegane [86].

Another example of the use of lipase regioselectivity to obtain APIs is represented in Scheme 1, where mono-hydrolysis is allowed to obtain a precursor of (+) isostegane [86] from a prochiral substrate (see Enantiomeric/Prochiral Preference (Regioselectivity in Chiral Triglycerides)). 

In summary, in processes where regioselectivity is sought, the structural and chemical restrictions of each enzyme, substrates and the particular dynamics of the catalytic phenomenon must be taken into account. This includes the adsorption of the enzyme at the interface constituted by insoluble substrates and the thermodynamic parameters of the reaction. Together, these complexities have made it difficult to establish universal relationships between structure and regioselectivity in lipases with their natural or synthetic substrates [103], thus constituting a knowledge gap to be solved in the subject. 

#### Enantiomeric/Prochiral Preference (Regioselectivity in Chiral Triglycerides)

Undoubtedly, finding chemical or physical processes that favor the enrichment of products with defined chirality and high purity is a continuous operation in synthesis laboratories and in the pharmaceutical industry. As is known, the guaranteed high purity of chiral active principles for drugs implies greater safety for the consumer. Once the intensification and optimization of the process to obtain those APIs has been achieved (with the consequent reduction in costs and environmental impacts), the sustainability of the pharmaceutical industry will be strengthened [35,37], which is significant considering that this industry is one of those facing the greatest challenges to comply with the principles of green chemistry [36]. 

Due to their advantages, microbial lipases have had a leading role in the solutions to these challenges through the different reactions that they catalyze by favoring reaction paths that lead to processing substrates and obtaining products of defined chirality through strategies such as kinetic resolution [12,114]. This selectivity has been quantified through parameters such as the *enantiomeric excess* percentage (*ee%*), which is the difference between the percentage of the major enantiomer and *percentage* of the minor enantiomer produced in a reaction mixture*. It is related to the enantiomer* ratio *(E)*, which depends on the relative reaction rate of an enantiomer (in a racemate) to that of the other*,* using a biocatalyst under kinetically controlled conditions (e.g.*,* first-order or pseudo-first order regime*):* this reflects how much the biocatalyst stabilizes the transition state of a reacting enantiomer with regard to the other [115,116]. In the recent review by Chen et al., an exhaustive description of the structural origins of enantioselectivity in some lipases was made [117]. Here, we will summarize some of the general aspects highlighted in that reference, but they will be complemented by approaches not covered there.

**Figure 5:**
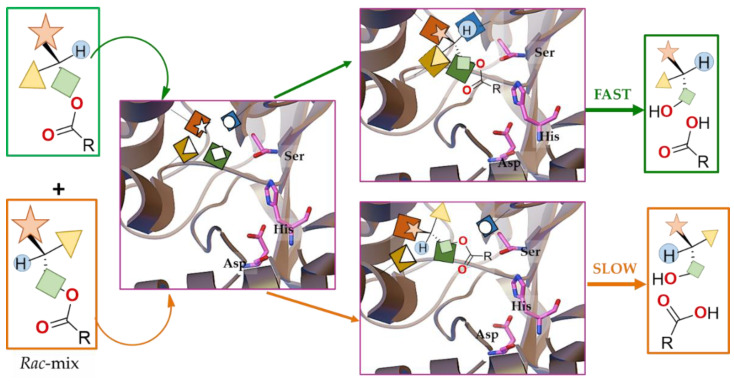
Representation of the “Mirror-Image Packing”: enantiomer recognition by enzymes relies on interactions between the substituents at the stereocenter and the recognition site. For a racemic mixture when the substrate has a stereocenter with -H as a substituent and is close to the carbonyl C, there will be a preference of reactivity to one enantiomer over another. For the enantiomer that react fast (green) all four interactions match, on the other hand, for the slow-reacting enantiomer (orange), only two substituents match (orange and green boxes) when all the substituents point to the interaction sites. Adapted from [117].

Structurally, lipases have two pockets in their catalytic cavity: one for the acyl group and one for the alcohol moiety in the case of an ester. The latter is distinguished by having a median and a larger pocket: the median pocket, also known as the “stereoselectivity pocket”, is located deep within the protein (in the case of CAL B), and the largest pocket constitutes the substrate entrance. Thus, a chiral secondary alcohol with substituents of different sizes (e.g.*,* methyl vs. propyl or bigger) can be sterically differentiated [118], explaining why most lipases tend to prefer R-enantiomeric secondary alcohols (Kazlauska’s rule) [107]. As will be seen (Table 1), there are other factors that give rise to exceptions or different interpretations to this rule: the “Mirror-Image Packing” rule (Figure 5) has broad support based on crystal structures [119] or the “Key Region(s) Influencing Enantioselectivity”(KRIE), which is based on a review of papers by its authors [120] about mutant lipases that improved their enantioselectivity not only towards derivatives of chiral alcohols but also towards those of chiral acids. In the case of KRIE, they concluded that some characteristics are key when designing lipases with improved enantioselectivity, such as the distance between the chiral carbon and the carboxyl or alcohol function, as appropriate, and its interaction with lipase regions such as: (i) the acyl-binding pocket, (ii) the hydrophobic cavity, (iii) the hydrophilic cavity and (iv) the oxyanion hole [120]. However, to generalize the KRIE, greater experimental support is required, something that will only be achieved by testing it with a greater number of lipases, substrates and reaction conditions.

In conclusion, the enzyme and substrate structure are dynamic variables due to their mutual interaction or due to the reaction conditions during the catalytic process: it is not always possible to attribute the observed selectivity nor to design strategies to improve them based on first principles or by taking into account a single factor. Thus, experience in the laboratory supported by an adequate experimental design will continue to be essential, not only as a criterion to refute or not the validity of the proposed structure-function hypotheses, but also as a source of new knowledge, sometimes unexpected. For example, it was observed that when using certain conditions, a lipase is capable of behaving like an enzyme of a different class, as described below.

#### 2.2.4. Reaction Specificity of Lipases

As the editor of this Special Issue mentions “the narrow specificity, and low promiscuity, or the high costs of enzyme production usually hamper the industrial applications of enzymes for APIs manufacturing”. Indeed, it is very difficult to ensure that the same enzyme maintains its selectivity (e.g.*,* high *ee*%) and a high turn-over number within the type of reaction it catalyzes regardless of the structural or chemical variants that the substrates may have. This is precisely due to the degree of specialization reached by some enzymes. On the other hand, as in the case with treatments against cancer or bacterial infections, the pharmaceutical industry is continually looking for new APIs, since those that were initially commercially available must be replaced, either because they have triggered therapeutic resistance, or because they must minimize its collateral or secondary effects [126,127]. One strategy to find new APIs is to generate modifications on a common chemical template to which the biological activity, known as pharmacophore, is associated. These modifications consist of, e.g.*,* introducing new substituents, positional changes, chemical functionalities, etc. For this reason, the enzyme that showed optimal properties for obtaining a building block of an API or the API that is intended to be replaced very probably will not be optimal for all the chemical and structural variants of the pharmacophore, which, in turn, will imply finding a new variant of the enzyme, either by prospection and/or by design, which, if not achieved in an adequate time, could entail replacing this biotransformation with a chemosynthetic step: the latter will normally be associated with a less green production process, something undesirable considering the very high E-factors that characterize this industry [36]. Of course, there is no single strategy to overcome these obstacles and to make finding biocatalytic variants that have, at least in part, the idealized properties for a catalyst needed in the production of a new API possible: computational models, molecular biology tools, enzymatic immobilization and reaction medium engineering, etc., provide tools to shorten the times in which that goal can be reached.

**Figure 6:**
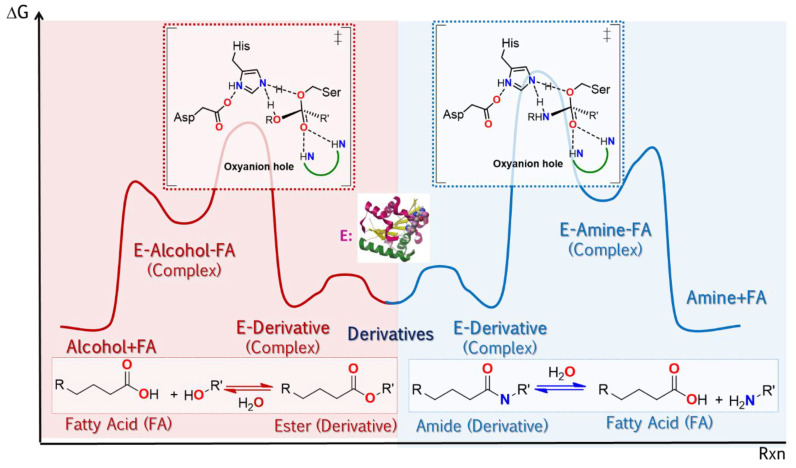
Schematic reaction coordinate vs. potential energy diagram for lipases, showing the transition state (‡) for each case. Representation of condition promiscuity: lipase can catalyze the hydrolysis of triglycerides (in the scheme, from the center towards the left), and under adequate circumstances, to catalyze reactions in the opposite direction (esterification). In addition, substrate promiscuity is present (**left**): they are capable of hydrolyzing other carboxylic acid derivatives such as amides or synthesize them from amines and carboxylic acids, here it can be seen that the difference between transition states (‡) is precisely the nucleophile involved. [133].

Another ideal situation for an enzyme to be attractive in industrial applications is that it is capable of presenting optimal properties, not only for the same class of reactions but also for others necessary to obtain an API. At the time, it was taken for granted that the catalytic domain of an enzyme could only catalyze some of the reactions of its class, for example, that hydrolases such as lipases could only carry out EC 3 class reactions. However, at least since 2003 [128], it has been shown that lipases such as CAL B are also capable of carrying out reactions previously assigned to the lyase class (EC 4). This property in an enzyme is known as catalytic promiscuity, although it is not the only promiscuity type (Figure 6). Next, we will focus on the molecular origins of promiscuity in microbial lipases, a topic covered more extensively in the articles of Kazlauzkas [129], Gupta et al. [31,78], Wang et al. [130] and recently for Dewivedee et al. [131] and Patti et al. [132], but with an emphasis on the way in which this property can be modulated so that this versatility may favor lipase application in industry.

**Figure 7:**
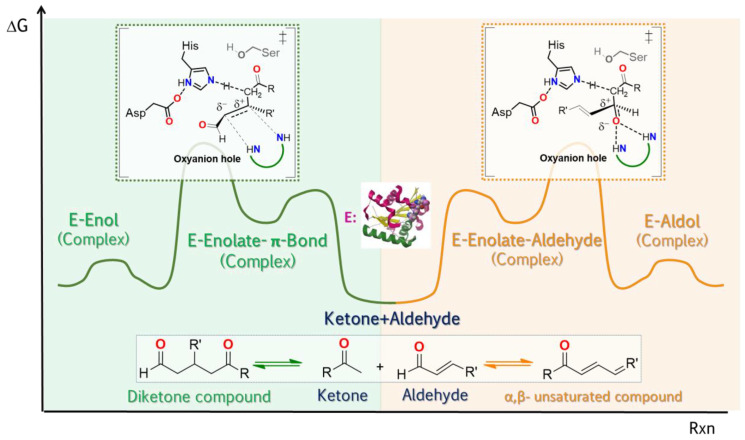
Schematic reaction coordinate vs. potential energy diagram for lipases. Representing an example of a catalytic promiscuity: the two-half panel, showing that the lipase can act in both directions, and product promiscuity: the enzyme as aldolase when faced with a substrate with two or more reaction possibilities, it is showed in each transition state (‡); as is the represented case of an unsaturated α, β aldehyde, the addition of ketone (in the form of enol) can occur in the β-position carbon or carbonyl carbon. One of the two products will be favored depending on which product favors the more stable conformation of the enzyme [135].

In addition to the three types of promiscuity previously exposed, a fourth type called “product promiscuity” has recently been considered [134]. This refers to when the same enzyme is able to convert a single substrate into multiple products in reactions that are independent of each other (Figure 7). In this sense, this means that for each reaction for the production of such products, it is necessary to go through different transition states [134]. Although the reactivity of the substrate functional groups have an effect on the regiochemics of the reactions, in particular for biocatalyzed reactions, it has been demonstrated that the conformation adopted by the enzyme prior to the formation of the substrate enzyme complex is that which determines the main product of the reaction, according to recent review [135]. Thus, depending on the stability of possible conformations, the regioselectivity of a reaction can be predicted. Complementary to this, in the same review [135], it details the importance and positive effect of conformational flexibility in a study on product promiscuity, using the native species of glutathione transferases and mutants with greater flexibility. It is important to recognize that although there are no reports of the use of lipase product promiscuity for the production of APIs, it must be recognized that this property can be exploited in the development of tandem methodologies towards the synthesis of APIs, where from the same substrate two precursors can be obtained that would react to each other to improve the possibility of obtaining the target molecule.

**Scheme 2:**
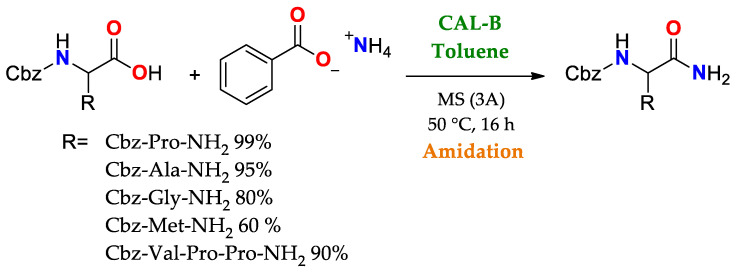
Use of CAL B in the amidation of acid ends of amino acid chains as a key stage in the synthesis of short peptides [136].

**Scheme 3:**
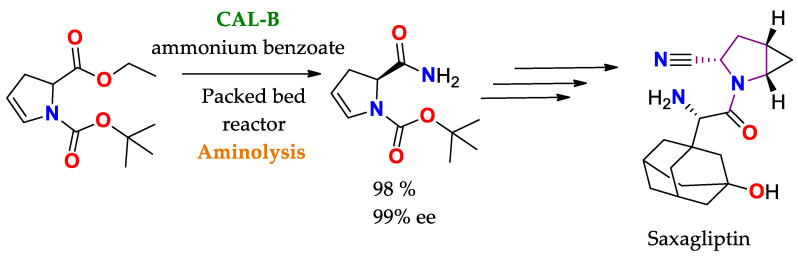
Selective aminolysis of 1-tert-butyl 2-ethyl 2,3-dihydro-1H-pyrrole-1,2-dicarboxylate to obtain a precursor of Saxagliptin (active drug ingredient for the treatment of diabetes) [136].

The broad uses of lipases as promiscuous catalysts takes advantage of its substrate promiscuity, such as in selective reactions involving the generation or cleavage of amido bonds. Some cases highlighted here are: i. amidation of amino acids and peptides using CAL-B, Scheme 2, and ii. selective transamidation to obtain a precursor of Saxagliptin (Active ingredient for some diabetes medications), Scheme 3 [136].

**Scheme 4:**
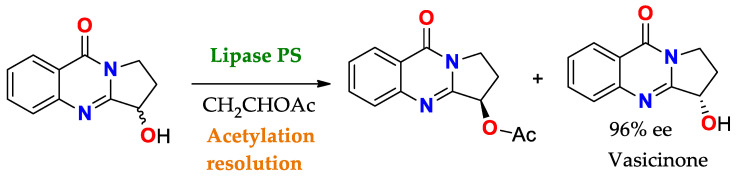
Acetylation towards the resolution of the racemic mixture of 3-hydroxy-2,3-dihydropyrrolo[2,1-b]quinazolin-9(1H)-one using PS lipase (Pseudomonas cepacia lipase) to obtain Vasicinone (molecule with bronchodilator activity and antianaphylactic action) [137].

**Scheme 5:**
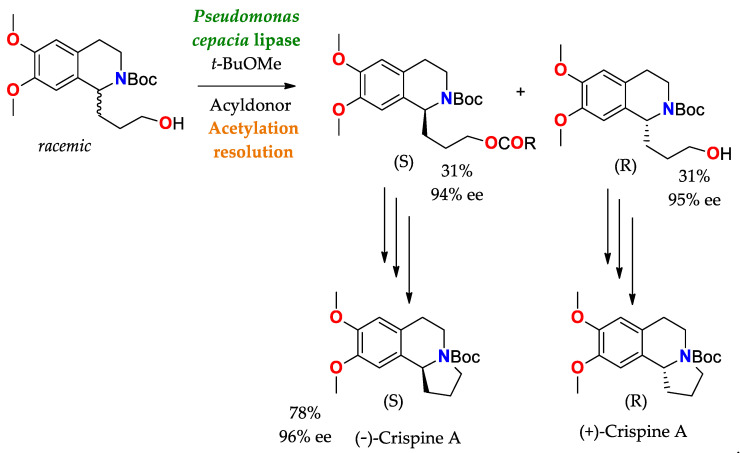
Synthesis and resolution of precursors of (−)−Crispine A and (+)−Crispine A (antitumor alkaloid), by means of acetylation using a lipase as a catalyst [138].

**Scheme 6:**
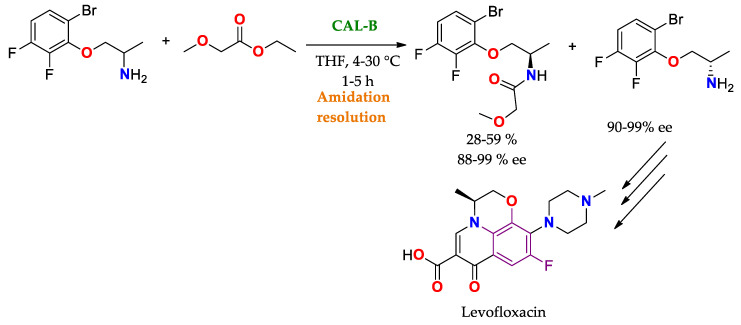
Obtaining (S)−1−(6-bromo−2,3−difluorophenoxy)propan−2-amine by resolution using CAL-B. Levofloxacin precursor, molecule with activity as an antibiotic [137].

This property has been used to obtain molecules with biological activity. In particular, in the resolution of racemic mixtures to obtain the final product (Scheme 4) [137] as for APIs resolution (Scheme 5 and Scheme 6) [137,138].

#### Molecular Origins of the Catalytic Promiscuity of Lipases

As mentioned, due to the nature of their substrates and the interfacial mechanism of action that some lipases present (Section 2.2), these enzymes are evolutionarily more prepared than others (such as esterases, proteases, etc.) to be functional in unconventional media and generally more hydrophobic than the aqueous medium. As Gupta indicates [78], this particular condition means that lipases can interact with substrates of a different nature (substrate promiscuity) through interactions mediated by entropy and are therefore less specific: this is caused by the wide hydrophobic area that constitutes the catalytic domain, including the lid in some lipases (Section 2.1 and Section 2.2). This contrasts with the interactions that occur in enzymes with smaller or more hydrophilic active centers in which polar or H bond interactions predominate and thus require more defined orientations and geometries from the substrate to form the Michaelis complex. 

On the other hand, lipases, being serine hydrolases (Section 2.2.2), possess an oxyanion hole that serves to polarize carbonyl groups present in their natural substrates through H bond interactions. This favors the attack of nucleophiles, including their catalytic Ser [133]. This polarization capacity is not only limited to interacting with carbonyl groups, but also to groups such as nitrile, which will be discussed later (Section 4.3) [33]. This oxyanion property makes it easier for the lipases to interact with substrates such as –C_α_-(C=O)- R, where R’ is not only an O atom (forming an alkoxy group) but also N or S, and even C or H, as in the case of ketones and aldehydes. Particularly, in cases when the catalytic Ser is removed using site-directed mutagenesis, the catalytic His can act base activate other nucleophiles when it removes their acidic hydrogens, not only from alcohols, amines or thiols but also from C_α_. The latter can be from a substrate housed in the oxyanion hole or from a substrate that is outside of it. This explains why the lipases can catalyze reactions as aldolic condensations or additions on α,β-unsaturated compounds (Figure 7) [133]. Significantly, lipases with these capacities are more advantageous than aldolases, as the former are more stable in non-natural conditions (they also have lower prices and broader commercial availability) [128], which is sometimes necessary in the case of organic synthesis reactions that involve obtaining API building blocks in which the substrates are insoluble in water or anhydrous media or high temperatures are required. Reactions involving lipase applications as a promiscuous catalyst are described in Section 4.

## 3. Immobilization of Lipases

The spatial confinement of an enzyme with respect to the reaction bulk is known as enzyme immobilization: it is considered a prerequisite for facilitating the application of enzymes such as lipases in the industry, as it facilitates their reuse by reducing costs, among other benefits [41,43,45,51,139]. The biocatalyst that results from the immobilization process of an enzyme, especially on solid supports, is often referred to as a “derivative”. As is expected, the immobilization process also involves a reduction in the configurational entropy associated with the unfolded state of the enzyme, resulting in greater stability (when compared with the free enzyme) against non-native conditions such as high temperature or the presence of organic solvents. This advantage of the immobilized enzymes promotes its application in the industry [82].

There are various ways to immobilize an enzyme. Immobilizing them on mesoporous and microporous supports has shown to be the preferred method in the industry, so in this section we will focus on this type of strategy [12]. As discussed in Section 4, a paradigmatic case of this type of immobilization has been that of CAL B supported on Lewatit^®^ VP OC 1600, known as Novozyme^®^ 435, a biocatalyst derivative very versatile given its uses in the industrial production of biofuels, pesticides, and, of course, APIs [12,140,141,142]. It is important to note that parameters such as the microstructure and surface properties of the support, enzyme, immobilization conditions (pH, temperature, ionic strength, presence of additives, etc.) and amount of immobilized enzyme, among others, should be optimized to the particular needs of the reaction to be catalyzed [41,44,51,85,143,144]. Some effects of the immobilization of lipases on meso- and macroporous supports within the production of building blocks of APIs are discussed below. 

### 3.1. Immobilization of Lipases and Effects on Their Properties in Reactions of Interest

#### 3.1.1. Immobilization by Hydrophobic Interaction

Immobilization by a reversible interaction such as hydrophobic and ionic, involves simple protocols and the possibility of support recovery and a reduction in costs in case the immobilized lipase loses activity during its application [43,44,145]. When the matrix or surface of the support is hydrophobic it has the advantage of “mimicking” that of the aggregated natural substrates on which the lipase acts in vivo as oils or fats (Section 2.2.1). This allows such supports to stabilize lipase open forms (catalytically more active) regarding those of lipases in the free state (Figure 2), something evidenced in studies with immobilized TLL on surfaces of oxidized graphene [146]. Additionally, when the immobilization procedure on hydrophobic supports is conducted in a medium with low ionic strength (and near to or at the pI of the enzyme), it becomes more selective by favoring interactions with proteins with broad hydrophobic domains on its surface such as lipases (lid, catalytic domain), implying the additional gain of purifying them while immobilizing [44,143,146]. It is important to note that in equal conditions in several studies, the specific activity for the enzymatic derivative in the hydrolysis of chromogenic esters (p-nitrophenylpropionate or p-NPP) generally exceeded the value against the free enzyme, with minimum increases of 1.5–3.0 times for BTL2 or up to 7.5–20 times for TLL. This was especially the case in derivatives with agarose matrix activated with hydrophobic groups (butyl or octyl), which accounts for the interphase activation suffered by lipase when immobilized. In addition, the fact that the immobilized lipases were completely desorbed by detergents not only supposes the regeneration of the support, but provides evidence for a reversible interaction by the hydrophobic effect between the enzyme and the support (Section 2.2.1) [44,147,148].

**Scheme 7:**
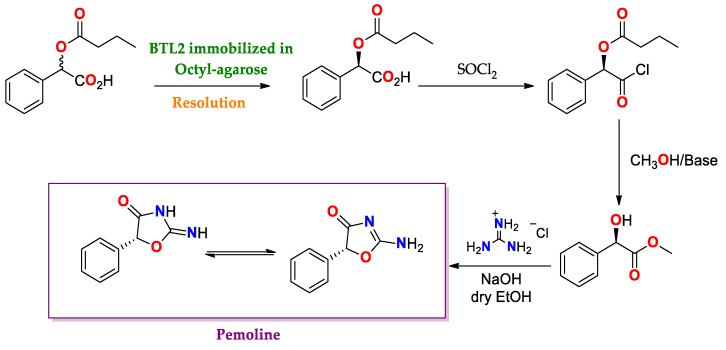
Proposed synthetic route using the resolution capacity of BTL2 derivatives with rac-2-O-Butyryl-2-phenylacetic acid for the synthesis of APIs ((R)-pemoline) [150].

One study evaluated the effect of the hydrophobicity degree of the support in which lipases BTL2, CAL B, TLL or phospholipase Lecitase^®^ were immobilized on in order to observe the effects on activity and selectivity in hydrolysis reactions [149]. In the case of a reaction with rac-2-O-Butyryl-2-phenylacetic acid (precursor of mandelic acid derivatives APIs as pemoline ([150], Scheme 7), homatropine, cyclandelate, cephalosporin, antineoplastics, antiobesity and anti-thrombotic agents [150,151,152]), BTL2 immobilized in octyl-agarose, butyl-agarose, butyl-Toyopearl^®^ or hexyl-Toyopearl^®^, showed preference for enantiomer (S), with values of the enantiomeric ratio (E) from 8 to >100, the latter for the octyl-agarose derivative [149] (Table 2). These observed increases by changing the support in E values are key for applications because, as a rule of thumb, values below fifteen are considered insufficient; from 15–30 moderate to good, and above this, excellent [153]. CAL B, TLL and Lecitase^®^ demonstrated less marked changes when immobilized in such supports; for example, the derivatives of CAL B changed their values of E from 3 to 60 for the derivative butyl-Toyopearl^®^ and octyl-agarose, respectively [149] (Table 2). 

**Scheme 8:**
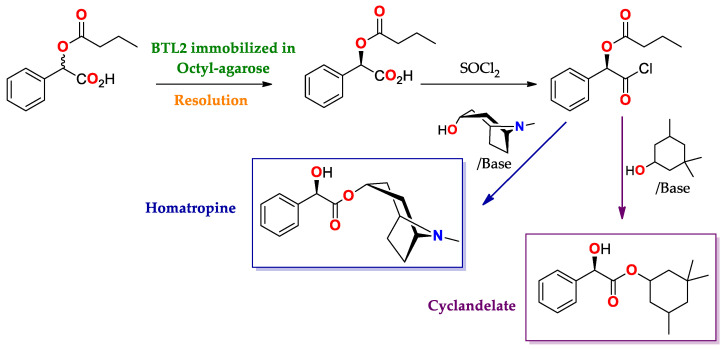
Proposed synthetic route using the resolution capacity of BTL2 for the production of the derivatives of mandelic acid [152].

Taking advantage of the results observed in the resolution of rac-2-O-butyryl-2-phenylacetic acid, here an extension of the use of the process in a synthetic route is proposed. It would allow the production of APIs derived from it (Homatropine (blue box), Cyclandelate (purple box) (Scheme 8). 

In the case of asymmetric hydrolysis of 3-phenylglutaric acid diethyl diester (precursor of drugs against HIV [147]) for BTL2 derivatives, desymmetrization factors “A” (usually the ratio of (R) to (S) products) increased from 3 to 116 towards the product (R), the latter being the derivative of hexyl-Toyopearl^®^ [149]. This type of desymmetrization reaction stands out for the possibility of obtaining optically pure precursors with a theoretical 100% yield without requiring the use of additional catalysts for racemization, something to take into account in the design of synthetic routes that are more efficient in the production of APIs.

In another study, the effect of different hydrophobic surfaces on the selectivity of CAL B derivatives against rac-2-O-Butyryl-2-phenylacetic acid were obtained: changes in the enantiomeric relationship of S (*E* > 100) to R (*E* = 49) were determined, passing from a support with a hydrophobic matrix (Lewatit^®^ VP-OC 1600) to one with a hydrophilic matrix (octyl-Sepharose^®^) [147] (Table 2). This same effect of the nature of the support caused an increase in values of *ee* (from 77% to more than 99%) for those derivatives in the case of asymmetric hydrolysis of 3-phenylglutaric acid dimethyl diester but without changes in the configuration of the product obtained (R) [147]. In the regioselective monoprotection of peracetylated glycopyranosides as the building blocks of lacto-N-neotetraose and the precursor of the tumor-associated carbohydrate antigen T and the antitumoral drug peracetylated β-naphtyl-lactosamine, octyl-Sepharose^®^ derivatives of CRL, TLL (Table 2), ANL y PFL were used. Selectivity was observed in the deprotection in C6 of 95% for the first two and 96% for the last two in C4 and C1, respectively [154].

**Figure 8:**
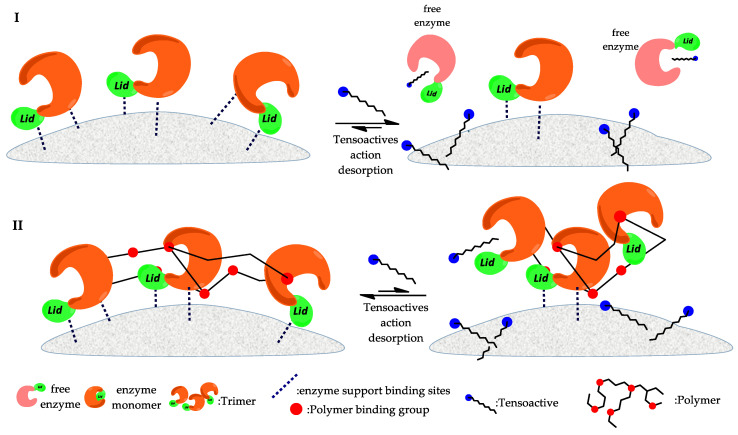
(**I**). Representation of lipases in monomeric form immobilized in their open conformation on a hydrophobic surface (represented in grey). In the presence of surfactants, some binding points to the support are lost, generating desorption of enzymes present in the derivative. (**II**) Representation of lipases immobilized in a hydrophobic surface crosslinked with an ionic polymer. In the presence of surfactant binding points are also lost, however here it is shown that in contrast to **I** there is no enzymatic desorption [161,162].

As mentioned, it is expected that immobilizations increase the thermostability of lipases, for example, free, CAL B lost 50% of its activity at 65°C between 1.4 and 3.0 h, while its hydrophobic derivatives did so only after 20–47 h, by contrast, stability in dioxane at 70% was similar to that of the free enzyme [149,155,156,157]. The thermal stabilization of the activity in the derivative against the soluble enzyme has the advantage of enabling biotransformations in the case of substrates requiring higher temperatures to increase their solubility, or when higher reaction speeds are desired; however, when an organic solvent has similar inactivating effects on the free enzyme to the hydrophobic derivative, it makes the latter unable to be applied when such solvents are required [44,145]. To solve this, strategies of physical or chemical modification of the hydrophobic derivative have been proposed such as the use of coatings with siloxanes [158], and other polymers such as dextran aldehyde [159] or polyethylenimine that can chemically or physically crosslink the immobilized enzymes [160]: this crosslinking protects the derivative from inactivation caused by desorption, since the enzyme will be part of a composite of higher molecular weight, in which more enzyme-support unions should be broken simultaneously to cause its release when compared to those in the case of the enzyme immobilized without modification (Figure 8). However, the possible gain in stability obtained in the derivative through these modifications is not only inevitably accompanied by an increase in the complexity of the immobilization protocol and the reuse of the support, it also involves changes in the activity or selectivity of the derivative, so the modification process should be optimized under those variables in a particular reaction [161]. This was evidenced in a recent study, in which the authors improved the stability and catalytic properties of Thermomyces lanuginosus lipase (TLL) adsorbed on a hydrophobic support by using additives that function as cross-linking units, allowing the loss of lipase to be reduced from 40% to <2% of the adsorbed enzyme, with only 0.5% *w*/*w* of the corresponding additive, further achieving in the case of CS the increase in catalytic efficiency by two-fold [162]. On the other hand, as an example of the use of these derivatives in API production, the PEI modification of an octyl-agarose derivative of the lipase Eversa^®^ in the hydrolysis of methyl rac-2-hydroxy-2-phenylacetic acid (also a precursor of cephalosporin, antineoplastics, etc. [151]), although it did not significantly change enantioselectivity, reduced the activity by 36% [159]. 

#### 3.1.2. Ion Exchange Immobilization

Like all polyelectrolytes, lipases at pH values far from their pI are likely to be immobilized by ion exchange on ionic support surfaces of an opposite charge to that of the enzymatic surface; however, the presence of Z potentials (ζ) of an opposite sign on the surface of the support and on that of the protein would be more determinant for the immobilization process, since this potential additionally depends on the ionic strength of the medium. Understanding this mechanism has direct implications for widely used enzyme purification techniques such as ion-exchange chromatography (IEX) [163]. On the other hand, unlike on hydrophobic surfaces, the orientation of the lipase on the charged exchange support surface will depend on the orientation of its net dipole moment vector: it points towards the surface of the support in the case of an anion exchanger or in the opposite direction in the case of a cation exchanger [80]. Due to the fact that in this type of immobilization, the interactions are reversible, similar to hydrophobic interactions, they have the potential to facilitate the recovery of the support from a derivative where the lipase has been inactivated. To do this, it is necessary to use solutions with high ionic strength or at a pH that gives the potential lipase (ζ) an equal sign to that of the support [163,164]. 

Considering the nature of the ionic interaction, it would be expected that the derivatives obtained were more resistant than the hydrophobic ones to the desorption of the enzyme from the enzyme in organic media. In this sense, hexane as an organic solvent, derivatives of CRL, with exchange resins in the resolution by transesterification of rac-menthol with vinyl acetate were used: the derivative with the cation exchanger Amberjet^®^1200-H had a yield of 45.2% and an E_(R/S)_ value of 208, while the anion exchange derivative Amberjet^®^4200-Cl showed yield values of 14.2% and an E_(R/S)_ value of 213 (Table 2). In contrast, the free enzyme showed yields lower than 3% and an E_(R/S)_ value of 68, probably due to inactivation promoted by the organic solvent [165]. The same lipase immobilized by anion exchange on Sephadex^®^ surfaces activated with diethylaminoethyl groups (DEAE) resolved rac-menthol in transesterification with valeric acid in hexane for at least 34 days of repeated use, maintaining 85% of its initial activity [166] (Table 2). Left-handed menthol derivatives such as (−)-menthyl chloroformate have been used in the resolution of enantiomers of the antiarrhythmic drug encainide and its major metabolites by chiral derivatization and high-performance liquid chromatography [167].

In another study [168], derivatives obtained by ion exchange using CAL B and PEI-agarose were applied in the resolution of methyl rac-2-hydroxy-2-phenylacetic acid (see relation to APIs in Section 3.1.1), these derivatives were obtained at different pHs and temperatures: the derivatives obtained at pH 5, 7 and 9 showed an E_(R/S)_ value of 3.5, 6.0 and 16, respectively. In that same study, when the immobilization temperatures varied between 4 °C, 25 °C and 37 °C, the E_(R/S)_ values were 25, 16 and 37, respectively (Table 2). Furthermore, CRL immobilized on DEAE-glycidyl methacrylate (GMA)-ethylene dimethacrylate (EDMA)-activated magnetic microspheres showed the highest activity when immobilized at pH 5.0 versus other pHs (*ee* > 90%, conversion 30%) in resolution by esterification of (±)-menthol with anhydride propionic in the hydrophobic ionic liquid 1-butyl-3-methylimidazolium hexafluorophosphate [169] (Table 2). This evidence suggests that differences in immobilization conditions (pH, T, etc.) generate different properties in the biocatalysts obtained; since they determine different conformational states in which the enzyme is immobilized, these can be memorized (bioimprinting), especially when the enzymatic derivative subsequently acts in non-aqueous media [169,170,171].

#### 3.1.3. Covalent Immobilization

This immobilization involves the chemisorption of the enzyme on the surface of the support through one or more binding points, which generally (but not always) constitutes an irreversible process. [41,172,173]. The chemistry of covalent immobilization is very varied. The most common involve the use of native nucleophilic groups on the protein surface such as the N-terminal, the ε-NH_2_ of Lys, ε^2^-NH of His, β-SH of Cys or OH (Ser, Thr or Tyr), which play the role of nucleophiles that react with electrophilic groups on the surface of the support such as epoxides, vinylsulfone, disulfide, cyanate ester (CNBr), aldehydes, etc. On other occasions, the reaction mechanism may involve the formation of free radical-type intermediates, as is the case of the reaction between β-SH groups and alkene on the support in the presence of an initiator (thiol-ene reaction) [174]. It should be noted that the diversity with which this immobilization phenomenon can occur (mechanisms, immobilization conditions, etc.) can be increased when non-natural groups are added to the enzymatic surface (O-acylisourea, dienophiles, diene, alkyl-amino, alkylazide, alkyne, etc.) or when new functionalities are added to the surface of the support (multifunctionality) in order to favor or complement the enzyme-support covalent interaction through hydrophobic or ionic interactions, among many others [41,175,176,177,178,179,180,181,182].

**Figure 9:**
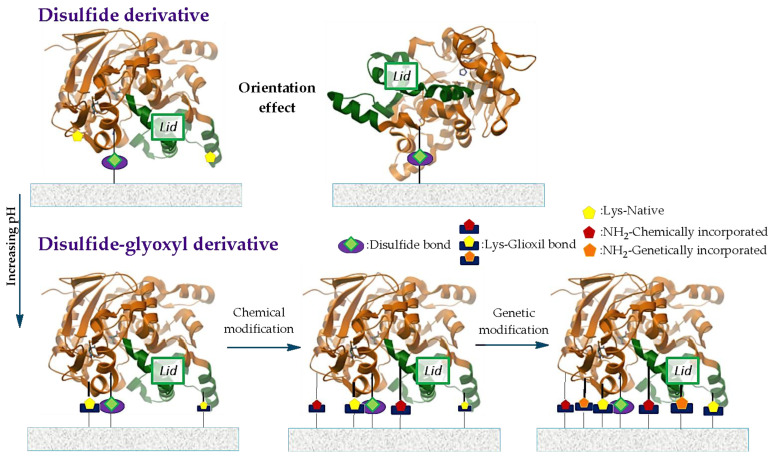
Control of enzyme orientation and binding intensity in glyoxyl/disulfide supports. Disulfide bonds are produced using Cys (Green) residues (introduced by site-directed mutagenesis) allowing oriented enzymatic immobilization in disulfide and glyoxyl-disulfide supports at pH 7.0. In addition, if after that the pH is increased glyoxyl groups (blue) will react with the Lys-native residues (yellow). On the other hand, the bonding degree can be increased by incorporating new anchor groups (NH_2_) through: (i) Chemical modification: addition reactions of EDA (ethylenediamine) to the carboxylate groups of the Asp and Glu residues. (ii) Genetic modification: changes in the protein sequence from Arg to Lys residues introduced by site-directed mutagenesis. All the above allows achieving control of the orientation and degree of binding of the enzyme.

Covalent immobilization on supports with glyoxyl groups (alkoxyacetaldehyde) have highlighted several advantages of the respective enzyme derivatives [178]. Glyoxyl supports allow lipases such as BTL2 or TLL to be hyper-stabilized under conditions such as high temperatures and the presence of organic co-solvents in magnitudes that exceed molecular biology tools such as directed evolution oriented for that purpose, as a result of the multipoint covalent union promoted by these supports [173,177]. Another advantage of glyoxyl-supports, such as those observed in derivatives of BTL2 and TLL, is that they are able to be reactivated using unfolding–folding strategies through the use of chaotropes, cosmotropes and other additives [47,48,49]. In these type of supports, it has also been possible not only direct the orientation of monocysteine-type designed variants of BTL2 (and penicillin G acylase), but also the intensity of their union on the surface of the support, something not so simple using immobilization strategies such as ionic or hydrophobic [183,184]. This has added to evidence regarding how different orientation and intensity of binding in the same lipase (BTL2) can generate changes in selectivity in reactions for the production of APIs, such as in the kinetic resolution of rac-2-O-butyryl-2-phenylacetic acid and in the desymmetrization of the phenylglutaric acid diester (Table 3 and Figure 9) such as APIs building blocks (Section 3.1.1 and Scheme 7). As seen in Table 3, the changes caused by the orientation and intensity of the lipase binding in the covalent derivative are less marked in the selectivity than in the thermal stability of the biocatalyst: this is a consequence of the reduction in the entropy in the denatured state generated by the binding of the enzyme to the support through covalent bonds; the more bonds there are, the greater the reduction [82]. This is consistent with another study where CAL B was covalently immobilized by varying the spacer arm (which defines the length of the enzyme-support bond) and observing effects on the transesterification of rac-1-phenylethanol with vinyl acetate: regardless of the length of the spacer arm, almost all of the derivatives had a preference for (1R)-1-phenylethanol (*ee*_(R)_ > 98%; E(R) = >200).The differences observed were mainly in its resistance to desorption caused by detergents and in the operational stability [185].

#### 3.1.4. Comparisons between Types of Immobilizations

The intensity (number of binding points and bond strength) in the enzyme-support interaction, as mentioned, favors the stabilization of the enzyme as a consequence of negative entropic effects in the denatured state [82]. In the native state, these unions generate greater enzyme rigidity, which leads to a widening of the optimal ranges of activity against, for example, pH and T. In the case of the latter, a displacement of the optimal ranges is observed at higher temperatures [41,173,178]. This explains why, in particular, multipoint covalent derivatives usually present the highest degrees of stabilization when compared to derivatives of a reversible nature such as ionic and hydrophobic ones [177,178]. In molecular dynamic (MD) simulations, CAL B showed an interaction of −632 kJ/mol with a hydrophobic surface, −106 kJ/mol with a cationic surface, and −346 kJ/mol with an anionic surface [80]. In the case of covalent derivatives, if the enzyme is linked through more than two C-N bonds, which is common in derivatives such as glyoxyl or glutaraldehyde supports, the interaction achieved would already exceed −900 kJ/mol [178]. Despite this, the stability of the enzymatic activity observed in the derivatives does not always seem to follow the energy trends mentioned, being clear that this will depend on many factors: the type of support used, the inactivation conditions, the type of substrate used in the measurement of activity and the exact orientation of the enzyme on the support. This is due to its heterogeneity in each region of the enzymatic surface involved in the binding with the support, which will have a differential stabilizing effect [183,186]. For example, when the lipase from Hypocrea pseudokoningii was immobilized on a hydrophobic support, the time required to loss of 50% of the initial derivative activity at pH 7.0 and 60 °C was at most 2 h (octyl-Sepharose^®^), but in the derivatives based on ion exchangers was superior, achieving times of 4–24 h (Q-Sepharose^®^ and PEI-agarose, respectively). In this study, different solvents at 50% (*v*/*v*) were also tested, such as methanol, ethanol, propanol and cyclohexane. In general, in methanol, there were no notable differences between derivatives that achieved the lowest or highest stabilizations, regardless of whether they were based on ionic or hydrophobic supports (4–24 h). In propanol, ethanol and especially in cyclohexane, hydrophobic supports always generated the highest stabilizations; for example, in cyclohexane, most ion exchangers (DEAE-agarose, Q-Sepharose^®^ derivative, MANAE-agarose, PEI-agarose derivative and Duolite^®^) lost about 50% of activity at 48 h, while the hydrophobic ones (butyl-Sepharose^®^, phenyl-Sepharose^®^, octyl-Sepharose^®^, hexyl Toyopearl^®^ and decaoctyl Sepabeads^®^) surprisingly increased their activity in that period up to 7 times [187]. In another study [188], there were no notable differences in stability at 90 °C or after 4 h in 50% THF between the ionic derivative Q-Sepharose^®^, the hydrophobic octyl-Sepharose^®^ or the hydrophobic-covalent derivatives octyl-glyoxyl-Sepharose^®^ for the lipase from Psychrophilic Serratia sp. (USBA-GBX-513). Using the same supports for CAL B but with the addition of glyoxyl-agarose, a half-life activity time of 192 min was observed for the latter, 240 min in octyl-glyoxyl-Sepharose^®^ and 144 min in octyl-Sepharose^®^, all in dioxane at 80% and 30 °C. This is evidence of how a different orientation of the enzyme (area richer in Lys for glyoxyl-agarose or a more hydrophobic area for octyl-Sepharose^®^) or the addition of additional covalent bonds within the same orientation (octyl-glyoxyl-Sepharose^®^) modulates and improves the stability of the biocatalyst [189].

On the other hand, in some applications that involve recovering the lipase from the support used, either for its purification or to carry out a chemical modification in the solid phase [170,190], there are differences to consider between ionic or hydrophobic exchange supports (covalent ones are ruled out as they mostly imply irreversible unions). In the former, the use of a buffer at an adequate pH and with a high ionic strength should be sufficient to desorb the lipase. The excess of salts used for this purpose can be easily removed from the enzyme solution by techniques such as dialysis, ultrafiltration or size exclusion. In the case of hydrophobic ones, the use of detergents for their desorption is frequent, which makes it difficult to remove their excesses from the enzymatic solution by the techniques described, or where the use of polystyrene beads for this purpose is not an option since the lipase could also be co-immobilized on these [191,192]. However, the most outstanding advantage of supports that immobilize lipases by hydrophobic interaction is that they “mimic” the natural surface conditions in which lipases are usually more active (Section 3.1.1). The ionic ones usually require immobilization pH values where the enzymes are not necessarily more active or stable, for example, if an enzyme has an acidic pI, even more acidic pHs will be required to favor its immobilization in a cation exchanger, which could trigger its inactivation. In the case of covalent derivatives, immobilization conditions such as the use of cross-linking agents such as glutaraldehyde [180,193,194] and carbodiimide type activators [195], or the need of alkaline pHs and hydride-type reducing (agents used in glyoxyl immobilizations), could affect lipase activity [177]. This also implies immobilization processes that involve more steps and higher costs, in short, are less green than the other types of immobilizations [189,196]. Despite this, covalent or ionic supports would have the advantage of producing derivatives where enzyme desorption is reduced to zero or minimized, avoiding contamination of the product, even when reaction media implies the use of organic or non-conventional solvents, which are often required to favor synthesis reactions, promiscuous catalysis (Section 2.2.4), or favor the dissolution of substrates that are poorly soluble in water. As already indicated, when the desorption of the lipase from hydrophobic or ionic derivatives occurs due to the effects of the reaction conditions, it is necessary to carry out chemical modifications on the enzyme (Section 3.1.1) or on the support to prevent it [189].

There are a few studies in which the effects of the nature of the three types of immobilizations on the activity and enzymatic selectivity of reactions are analyzed. In one study, immobilized CAL B was used to act on the resolution, by hydrolysis, of derivatives of rac-5-substituted-6-(5-chloropyridin-2-yl)-7-oxo-5,6-dihydropyrrolo[3,4b]pyrazine as precursors to (S)-(+)-Zopiclone (used to treat insomnia) [197]. For this, the covalent supports CNBr, glutaraldehyde and glyoxyl, the PEI anion exchanger, all in an agarose matrix, and the hydrophobic support octadecyl-Sepabeads^®^, were used. When the vinyl carbonate derivative-type substrate was used at a concentration of 0.05 mM, all the supports showed an E_(R)_ > 100; the most noticeable differences were in terms of the specific activity that ranged between 1.0 and 2.5 IU/g of support, being the lowest for the glyoxyl derivative and the highest for the CNBr derivative. In order to increase the efficiency of the process, it was sought to increase the substrate concentration to 10 mM, but for this, 50% dioxane had to be used, given the low solubility of the substrate, the CNBr and PEI derivatives had to be discarded due to their lower solvent stability: the specific activity values for the octadecyl-Sepabeads^®^ derivatives, glutaraldehyde-agarose and glyoxyl-agarose were similar (1.20 IU/g to 1.56 IU/g), while the *ee* values (%) were >99.97 and 94, respectively. Considering these results, the derivative chosen to test other substrate variants was octadecyl-Sepabeads^®^, which also showed high stability in at least 10 reaction cycles without significant loss of activity or selectivity [197]. The same biocatalysts were tested in the hydrolysis of derivatives of rac-1-hydroxy-phenylacetic acid, useful in the synthesis of cephalosporin antibiotics such as Cephamandole and Cephonicid [198]. With the methyl ester, the PEI-agarose derivative was the most active (60 IU) and the CNBr had the least activity (1.47 IU) and the most enantioselective derivative was that of PEI-agarose (*ee*_(R)_ = 91%, E_(R)_ = 67). The one with the lowest selectivity was the CNBr-agarose derivative (*ee*_(R)_ = 60%, E_(R)_ = 7.4). In the case of the 2-O-butyroyl derivative, all lipase derivatives were between 3 and 5 orders of magnitude less active than with the methyl ester, the most active (0.0318 IU) and enantioselective being the covalent derivative glutaraldehyde-agarose with *ee*_(R)_ = >99.5% and E_(R)_ > 400. In another study [199], the derivatives of the lipases CAL B, CRL and ANL in octyl, PEI and CNBr, all in an agarose matrix, were evaluated in the regioselective monohydrolysis of per-O-acetylated 1-O-substituted-β-galactopyranosides to obtain blocks of construction for the glycosylation of antitumor drugs such as erythromycin or vancomycin [200]: ANL lipase showed the highest immobilized hydrolysis activities on PEI-agarose, CAL B on CNBr-agarose and CRL on octyl-agarose. Regarding regioselectivity, the lipases CAL B and ANL tended to carry out mono-hydrolysis at positions C-1 and C-6, but the type of immobilization had no effect on regioselectivity, which was low. In cases where the substrate had the thioisopropyl group instead of the acetyl group (previous case) at C-1, the regioselectivity towards C-6 increased markedly, being higher for ANL on the CNBr support and for CRL on the octyl support (CAL B was not tested). With 3,4,6-tri-O-acetyl-D-galactal (to 1,2-anhydrosugar), CAL B was not very regioselective, although it preferentially hydrolyzed C-3 regardless of the derivative and ANL had a similar behavior, although with preference in hydrolyzing C-4. CRL presented the highest regio-selectivity in octyl-agarose, obtaining almost exclusively the monohydrolyzed product in position C-6. 

Regarding recent patents, one complete article reviews the use of lipases [12], some immobilized in the production of APIs. Table 4 below summarizes some of the most relevant protected applications that highlight the advantages of using immobilized forms of lipases.

**Scheme 9:**
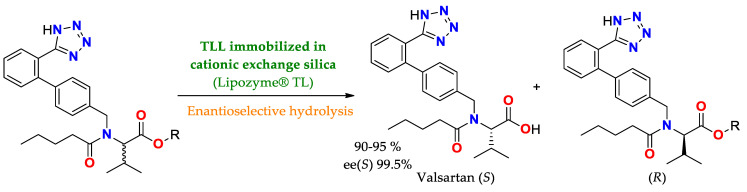
Obtaining Valsartan (antihypertensive) by hydrolysis resolution from a racemic mix using TLL immobilized [201].

**Scheme 10:**
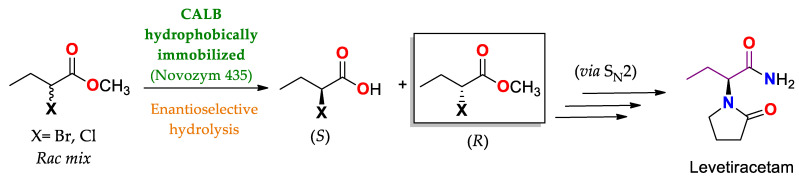
Obtaining a precursor of Levetiracetam (anti-epileptic drug) by hydrolysis resolution from a racemic mix using CAL B immobilized [12].

In general, regarding the use of enzymatic derivatives, the inherent selectivity of lipases in the resolution of racemic mixtures was used to obtain active compounds directly or precursors thereof (Scheme 9 and Scheme 10) [12,201]. 

**Scheme 11:**
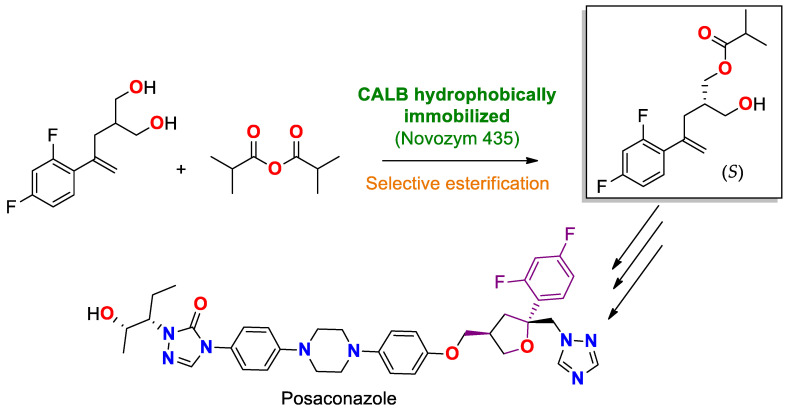
Obtaining a precursor of Posaconazole (anti-epileptic drug) by selective esterification from a no chiral substrate using CAL B immobilized [12].

**Scheme 12:**
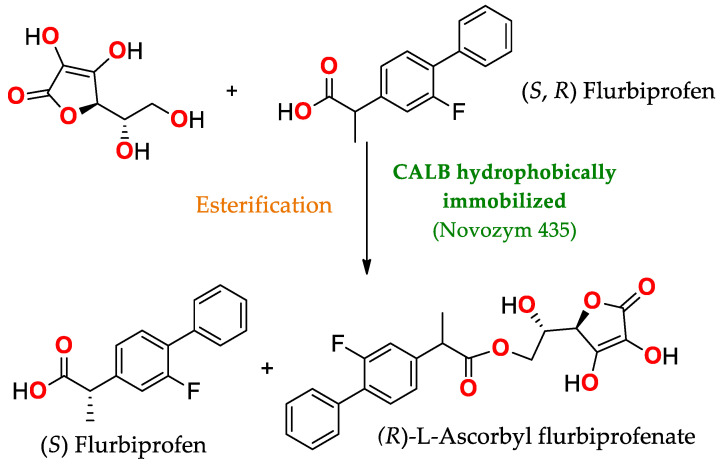
Obtaining a (R)-L-ascorbyl flurbiprofenate by esterification of a racemic mix of flurbiprofen and ascorbic acid using Novozym 435 [113].

In addition, the exploitation of the inherent hydrolytic capacity of lipases also takes advantage of the conditional promiscuity of lipases (Figure 6). The following are some examples of the use of immobilized lipases in key esterification processes in the synthesis of biologically active compounds (Scheme 11 and Scheme 12) [12,205]. 

In conclusion, the immobilization of lipases is a strategy that enhances the stability, possibility of reuse and reactivation of the generated biocatalysts. It also allows modulating properties (conformational engineering) such as its selectivity activity in reactions of interest in the production of APIs. However, there are still no predictive tools due the complexity of the systems involved that allow establishing which will be the best types of supports, immobilization conditions and lipase for a reaction of interest under particular conditions, so it is through experimental study that such responses will be established.

## 4. Applications of Free or Immobilized Lipases Related to APIs

Some of the reactions catalyzed by lipases in “promiscuous” mode were decrypted here (Section 2.2.4). Most corresponded to *substrate promiscuity*; that is, acyl group transfer to alcohols or amines, which, as shown, have applications in the synthesis of APIs. This section will describe the reactions catalyzed by lipases showing catalytic promiscuity, whose potential for application in the synthesis of APIs is, in many cases, a fertile ground for exploration on a more commercial level, given the lipases in regard to other types of enzymes (see molecular origins of promiscuity and benefits of lipases in Molecular Origins of the Catalytic Promiscuity of Lipases).

### 4.1. Additions on Carbonyl Compounds: Lipases Substituting Lyases

Among the first evidence of catalytic promiscuity in lipases is its action in aldol and Michael addition reactions where C-C bonds were formed in carbonyl compounds [128]. These type of reactions catalyzed by lipases along with Mannich reactions (multi-component) were recently reviewed by Patti et al. [132]. Here, we will focus on lipase applications in *aza*-Michael addition reactions, the Knoevenagel condensation reaction [30,206], among others, in connection with the production of APIs. 

#### 4.1.1. Aza-Michael Addition Reaction

**Scheme 13:**
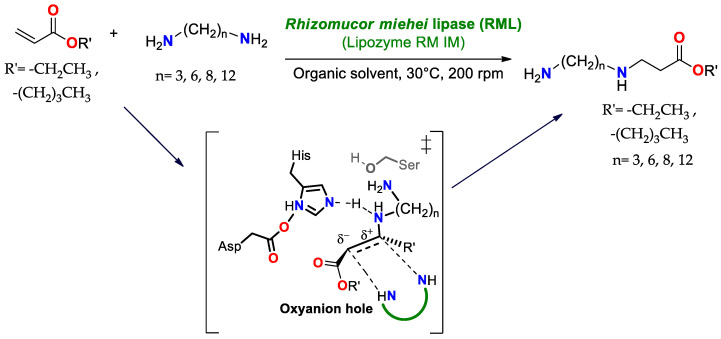
Addition of *aza*-Michael catalyzed by *Rhizomucor miehei* lipase RML (here, proposing a transition state) for the synthesis of heteropolycyclic precursors [207]. In the transition state (‡) it is shown that the nucleophile adds to the beta carbon favored by the activation of: 1 The His residue increasing the negative charge density of the nitrogen. 2 the oxyanion hole activating the electrophilic carbon (β-carbon).

A derivative lipase of *Rhizomucor miehei* (RML), Lipozyme^®^ RM IM, was applied in *aza*-Michael-type addition for the synthesis of β-amino esters as a precursor of bioactive heterocyclics or a polymer for gene delivery with potential use in gene therapy [207,208]. RML derivative was able to form in each case of Michael’s monoadduct as the only product with a high yield and purity (45–100%). Additionally, when diamines were used as nucleophiles, the lipase was able to perform regioselectively (Scheme 13) [207]. 

In addition to regioselectivity, another advantage of the lipase derivative used is its ability to be modulated without becoming inactive in the course of the reaction; even in the presence of low polarity solvents that are required to favor Michael’s addition, such as hexane, toluene and diisopropyl ether (DIPE), it still remained in the temperature range of 30–55 °C. It is noted that no study on the reusability of the used derivative has been carried out.

A similar approach (*aza*-Michael addition type reaction) in another later study used the free CAL B lipase in the chemo and stereoselective one-pot synthesis of (*S*)-β-amino esters with high *ee* (>95%), which is a potential building block of APIS as antibiotics but also in building blocks of chiral auxiliaries and agrochemicals [208]. Again, the reactions required working on low polarity solvents such as toluene, THF or acetonitrile at 50 °C to obtain the highest yields, the inherent viability of CAL B being key in enabling such processes.

On the one hand, the lipase’s ability to be stereoselective, acting in acyl transfer reactions (*conditional promiscuity*), and on the other hand, its ability to form C-N bonds (*catalytic promiscuity*), stand out in order to obtain products of greater complexity and high optical purity.

#### 4.1.2. Knoevenagel Condensation 

**Scheme 14:**
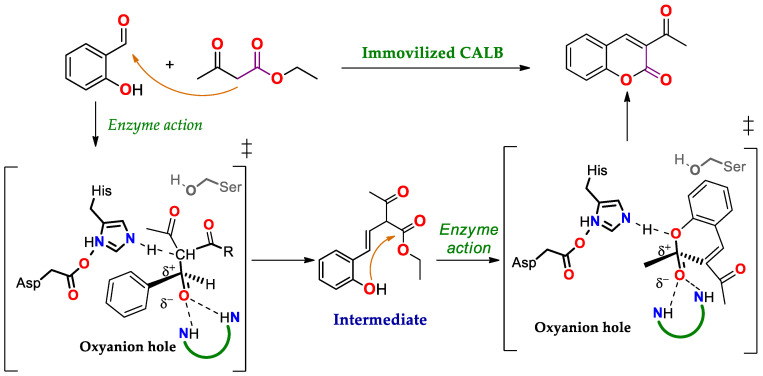
Proposed mechanism including possible transition states of Knovenagel condensation for the synthesis of benzopyran pharmacophore, present in APIs such as Bergapten (anti-inflammatory) or Methoxsalen [209]. Here the product is obtained by two addition steps. In both cases it is represented how the enzyme promotes the reaction, in the transition states (‡) the Asp and His residues are shown, allowing the activation of the nucleophile and the oxyanion hole the stabilization of the electrophilic carbon to which it is added.

It was found that free CAL B [206], immobilized covalently [30], is capable of catalyzing nucleophilic compound addition reactions with active hydrogen in C_α_ to a carbonyl group, followed by dehydration. The covalent derivative of CAL B was compared with its free form in the reaction between salicylaldehyde and ethyl acetoacetate (Scheme 14) for a drug containing benzopyran, present in APIs such as Bergapten (anti-inflammatory) or Methoxsalen [209] (psoriasis treatment) and Nebivolol (β-blocker antihypertensive) [210], among others. Under optimal conditions, the derivative achieved yields of up to 73% in a methanol mixture: water 4:1 to 60 °C for 5 h. Under these harsh conditions, the covalent derivative of CAL B was at least 3 times more stable, with the advantage of being able to be reused for at least 5 reaction cycles.

**Scheme 15:**
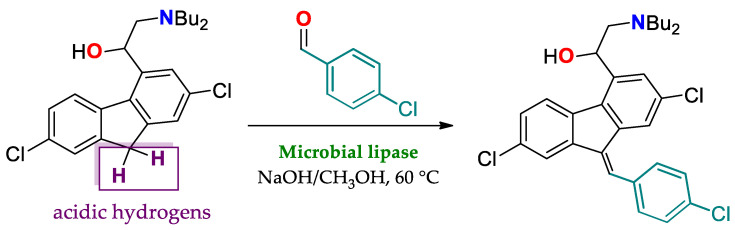
Lumerfaritine animal synthesis proposal (part of Coartem), by means of a Knoveneagel reaction catalyzed by microbial lipases. Adapted from [211].

The application of lipases in Knoevenagel condensations could also be of interest when obtaining APIs such as the lumerfaritine animal (part of Coartem), so that the Z isomer is obtained, preferably (Scheme 15) [211]:

#### 4.1.3. Reaction of Morita–Baylis–Hillman (MBH)

**Scheme 16:**
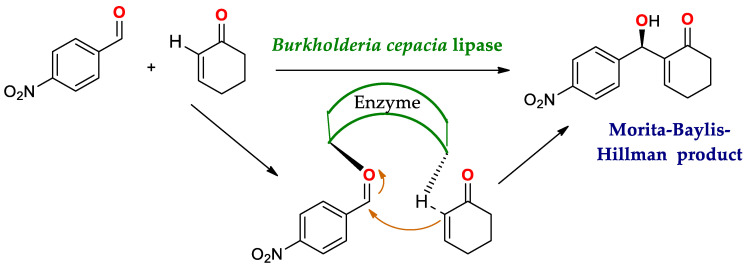
Representation of the use of the lipase *Burkholderia cepacia* as promoter of the stereospecific Morita–Baylis–Hillman reaction [31], notice that the addition must occur on the *re* side of the enolate due to the chelation orientation to aldehyde provided by the enzyme.

The MBH reaction consists of the reaction of an alkene/alkyne activated in Cα that is added to an electrophilic carbon, giving a product generally referred to as the Baylis–Hillman adduct. This reaction has been recognized as one of the most useful in organic chemistry, as it combines aldol and Michael addition reactions in a single step, showing great versatility. It is also used frequently with organocatalysts [212,213]. In one study [31], *Burkholderia cepacia* lipase (BCL) was used within a stereospecific MBH reaction that competes with an aldol condensation reaction (*product promiscuity*) (Scheme 16). However, it was not possible to obtain the MBH product with a performance superior to 6%.

**Scheme 17:**
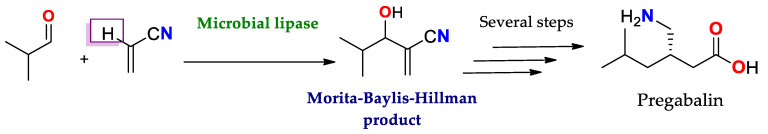
Proposal for the use of microbial lipases in the symptom of a key precursor in the synthesis of Pregabalin (antiepileptic). Adapted from [215].

Subsequently, the derivative Novozyme^®^ 435 (CAL B immobilized by hydrophobic interaction) was applied in the same reaction in the presence of amides (isonicotinamide) as a co-catalyst [214]. In this case, a yield of 43% to 37 °C was obtained after two days for the MBH product, although as a racemic mixture. In the absence of the lipase derivative or isonicotinamide, the yields did not exceed 9% (Scheme 17). This shows that the combined use of two types of catalysis (bio- and organo-) enables the generation of synergistic effects in reactions of interest. Therefore, applying these types of findings in MBH reactions in the production of APIs remains an area to be explored, for example, the products generated from the MBH reaction between 2-methyl propanal and prop-2-enenitrile could be used as a starting point in the synthesis of Pregabalin (anticonvulsant an anxiolytic) [213,215]:

#### 4.1.4. Kabachnik–Fields Reaction (KF)

**Scheme 18:**
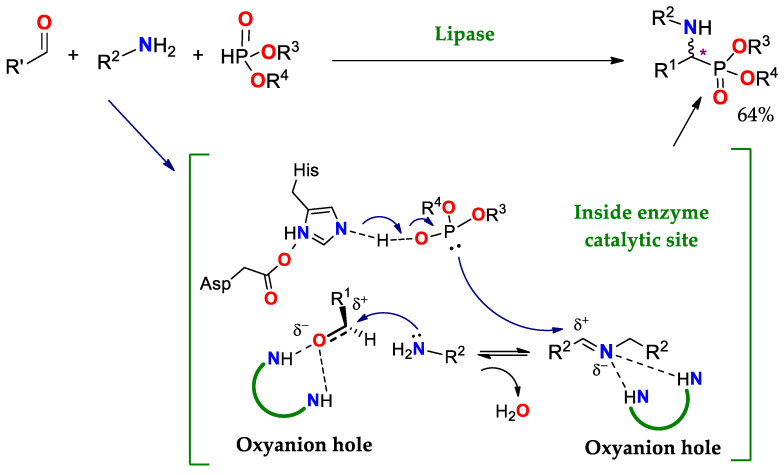
Proposed mechanism of the Kabachnik–Fields reaction using lipases. Adapted from [216].

The Kabachnik–Fields reaction is a multicomponent reaction in which α-amino alkylphosphonates are produced from a carbonyl compound, an amine and a dialkyl phosphonate (RO)_2_P(O)H (Scheme 18). In one study, the enzymes *Pseudomonas cepacia* lipase (PCL), *Candida cylindracea* lipase (CCL), and *Candida antarctica* B lipase immobilized on acrylic resin (CAL B) and the animal lipase Porcine pancreatic lipase (PPL) was evaluated in the benzaldehyde reaction, aniline and diethyl phosphonate at 25 °C and 24 h, achieving an optimal performance of 94% for immobilized CAL B. This produced the racemate. It was then tested for at least three reaction cycles, showing in the latter a yield of 64% [32].

In this sense, in the same research line, the enzymes from the previous study along with rabbit gastric lipase (RGL) were evaluated in the KF reaction using benzidine, diethylphosphite and several aldehydes in order to evaluate their diastereoselectivity in obtaining bis (α-aminophosphonates). CAL B immobilized in acrylic resin using THF as the solvent for 30 min and at 50 °C had a yield of 90% [217]. This enzyme derivative was reused for four cycles and in the last one, the yield was reduced to 69%. As for diastereoselectivity (meso/DL ratio), it depended on aldehyde used (electronic effects), but in most cases it was high (71 to 100%). These values were maintained when the synthesis scale was raised to the multigram order [217].

In a later study, the same lipases, along with Novozym 435, showed activities in the KF reaction between benzaldehyde, benzylamine and dimethyl phosphonate, with yields of 35–67% to 20 °C over 24 h [216]. The highest yield (67%) was obtained for Novozyme 435^®^ in biocatalysts based on microbial lipases; however, the highest was obtained with porcine pancreatic lipase (73% to 25 °C) [216]. Although many of the compounds synthesized in this study have chiral centers, the degree of optical purity was never detailed. Interestingly, different α-amino alkylphosphonates synthesized by this pathway were tested in terms of their antimicrobial activity, being derived with the presence of alkoxy, halogen or nitro groups in the most active phosphate function and, at the same time, the most cytotoxic (against different strains of *E. coli*) compared to common antibiotics, such as ciprofloxacin, bleomycin and cloxacillin [216].

It has been suggested that part of the biological activity of this type of α-amino alkylphosphonates could be due to the fact that they are structural analogs of α-amino acids, showing a low toxicity in mammalian cells, which supposes the great potential of their use as APIs [218], being the greener alternative catalyst lipases that, along with the use of microwave and solvent-free synthesis methods, could replace those based on heavy metals (Ir^3+^, Ni^2+^, Zr^4+^, Zn^2+^) or their MOFs [219].

#### 4.1.5. Lipase Acting in an Addition Reaction Type Markovnikov

**Scheme 19:**
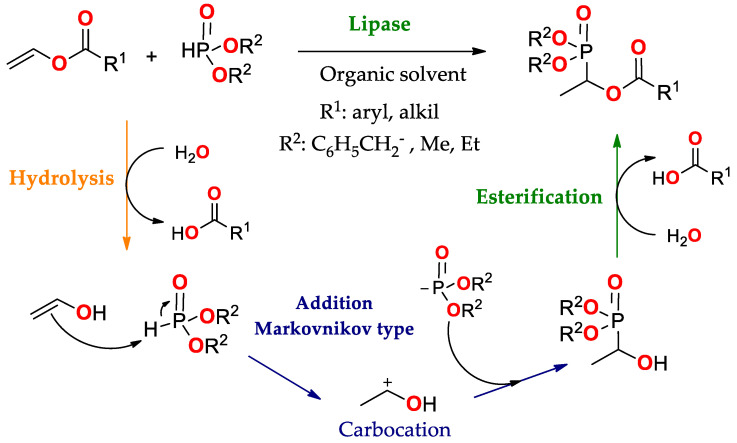
Synthesis of α−Hydroxy phosphonate derivatives using lipases as reaction catalyst. Here, a mechanism involving three reaction stages is proposed, where only the Markovnikov−type addition of the allyl alcohol double bond. Adapted from [220].

Recently, a series of α-Hydroxy phosphonate derivatives with potential application were synthesized using CCL by adding Markovnikov from H-phosphonates to vinyl esters (Scheme 19) [220]. Inside the α-Hydroxy phosphonate derivatives is the API Fosfomycin, a broad-spectrum antibiotic used to treat urinary infections without complications [221].

With CCL, the yield was up to 61% at 40 °C, and in n-hexane, other lipases such as CRL, PFL and the derivative of CAL B Novozyme 435^®^ showed lower yields (9–39%). This method of synthesis using lipases has the advantage of substituting catalysts based on heavy metals such as copper(II) trifluoromethanesulfonate or trichlorotitanium(IV) trifluoromethanesulfonate [220]. A library of enzymatically-obtained compounds were tested for their ability as an antibiotic against strains of *E. coli*, showing greater activity than common antibiotics, especially when dealing with α-acyloxy phosphonates with the methyl group at α [220]. 

### 4.2. Oxidation of Baeyer–Villiger: Lipases Substituting Monooxygenases

**Scheme 20:**
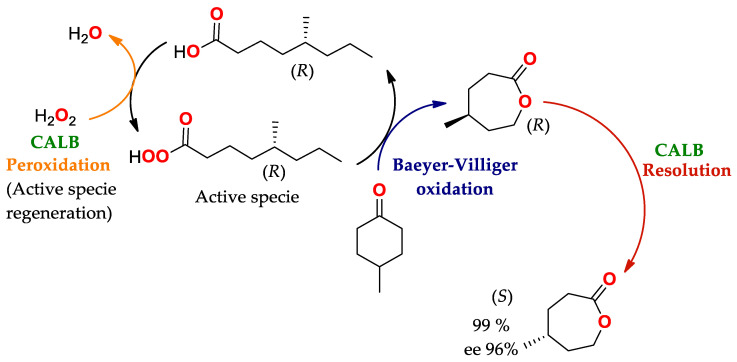
Chemo-enzymatic method for obtaining lactones by using Baeyer–Villiger oxidation: *Candida antarctica* lipase B (CAL B) participates in peroxide transfer, for the formation of the active species (peroxyacid) involved in the oxidation of Baeyer–Villiger of the ketone. Once lactone is obtained, CAL B is again involved in obtaining the enantiomer *S*. Adapted from [19].

In this case, *Candida antarctica* lipase B (CAL B) lipase was applied in the presence of H_2_O_2_ (aqueous) in the development of a chemo-enzymatic method in which Baeyer–Villiger asymmetric oxidation of prochiral ketone 4-methylcyclohexanone is performed to obtain (*R*)-4-methylcaprolactone. Here, the lipase transfers enantioselectively peroxide (acting as perhydrolase) to a racemic mixture of chiral carboxylic acid, generating an optical peroxyacid-active mind oxidizing cyclic prochiral ketone, leading to the formation of an optically pure lactone with a yield of more than 99% and *ee* 96% (*R*) (Scheme 20).

**Scheme 21:**
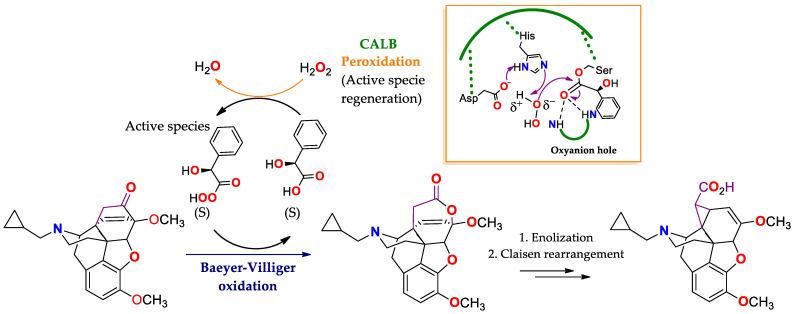
Proposed synthesis of κ opioid receptor agonists, by a lipase-catalyzed Baeyer–Villiger oxidation. Here, the lipase catalyzes the formation of the active species (peroxide) that is responsible for participating in the oxidation of Baeyer–Villiger to form a precursor necessary for obtaining the API by enolization and subsequent sigmatropic rearrangement of Claisen [224].

The advantages of the use of this lipase is to allow a green and cost-effective process that does not require cofactors such as flavin and NADP^+^, necessary in the case of Baeyer–Villiger monooxygenases [222], and to avoid the use of catalysts based on heavy metals (Cu^2+^, Sn^+4^), such as with those based on MOFs [19,223]. Extending the use of these lipase-mediated reactions could favor the synthesis of building blocks of APIs, as is the case of intermediaries for the design of κ opioid receptor agonists (Scheme 21), taking into account morphinan pharmacophore such as Narcan, which could be obtained by using this type of oxidation [224].

### 4.3. Racemization: A Lipase Substituting an Isomerase

**Figure 10:**
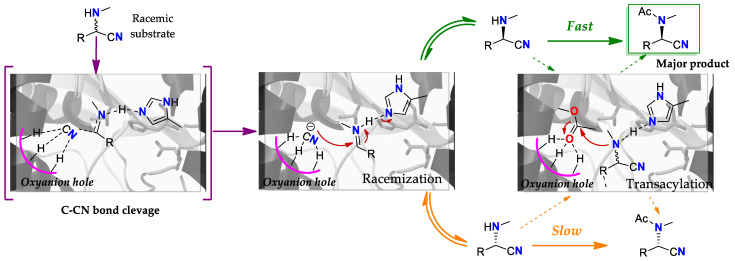
BCL as a dual biocatalyst. Acting as racemase of α-aminonitriles, facilitating the breaking and formation of the Cα-CN bond, in this way the two possible enantiomers are synthesized. In addition, the same BCL, acting as a transacylase, favors one of the two enantiomers, reaching an e.e of 85% adapted from [32].

Some approaches in the pharmaceutical industry regarding the development of new products imply taking advantage of an old racemic drug and marketing only its active enantiomer (chiral switch), or the development of a new but optically pure drug (*novo* manufacturing). In both cases, the application of the Dynamic Kinetic Resolution strategy may be a way to efficiently produce them [225]. When lipases such as CAL B (free or immobilized) have been used in DKR, they require an auxiliary catalyst with racemization activity against the substrate to be used. These usually require the use of an inert atmosphere in the case of a catalyst based on precious metals complexes [226]. This makes it harder to establish simplified and less expensive reaction systems required in the industry. Ideally, in the case of a lipase-catalyzed process, DKR would involve finding conditions in which the enzyme enantioselectively acylates/hydrolyzes a racemic substrate while favoring racemization of the enantiomer, for which it shows less affinity, so that eventually the yield of the reaction exceeds the conventional limit of 50%. To some extent, this has only been achieved with BCL lipase acting on α-aminonitriles, being able to break and form C_α_-CN bonds (as a racemase) and to preferentially transacylate one of the enantiomers, achieving an *ee*. of 85% at 40 °C in methyl *tert*-butyl ether, after 72 h of reaction (Figure 10) [33]. Generalizing this very interesting property to other substrates in greener conditions and under shorter reaction times will continue to be a challenge that, if overcome, will have a huge field of application in the API industry.

Finally, the above are just a few examples of the high versatility of microbial lipases acting as promiscuous catalysts in several types of organic reactions with applicability in the synthesis of building blocks of APIs. Research focusing on the customization of lipase catalytic promiscuity towards other substrates and reaction types, ideally under greener conditions and in shorter reaction times, will continue to be a challenge that, if overcome, will have a huge field of application in the pharmaceutical industry.

## 5. Perspectives and Conclusions

There are some aspects that must be considered to favor the still very incipient application of microbial lipases in the APIs industry [45]:

1. Studies on the environmental, economic and social costs (Life Cycle Assesment or LCA) with updated metrics that contrast the use of lipase-based biocatalysts against current synthesis strategies for specific APIs and the usual scale of production: of this there are examples in other industrial areas of application involving lipases and other enzymes [227,228]. Having well-defined cost–benefit ratios regarding the use of enzymatic processes in the industry is a solid starting point when overcoming obstacles to their implementation. This will allow us to prioritize what is worth investigating within what has not yet been done or take advantage of that which is still laboratory-scale or patent-protected but still unexploited.
2. The development of processes for the synthesis of APIs with the use of immobilized lipases, with the appropriate studies of reuse or continuous use, and, if possible, in the absence of organic solvents: in comparative studies of ACL of biocatalysis vs. conventional process, those two aspects are some of the most critical when favoring the implementation of enzymatic processes over chemicals in the industry [227,229,230].
3. The expansion of lipase promiscuity and the improvement of synthetic parameters such as reaction rates, performance and industrial-scale stereoselectivity: this requires the integrated use of tools such as directed evolution, enzymatic immobilization, enzyme chemical modification, QM/MM and, recently, Machine-Learning (ML) and Artificial Intelligence (AI) [231,232,233,234,235], applied to the old but also to the new microbial lipases that the required bioprospection can offer.
4. The experimental work will continue to be a source of knowledge in complex reaction systems such as those that normally involve immobilized lipases: an adequate experimental design for the generation of quality data will be indispensable to feed the improvement tools such as ML and AI.

Finally, although microbial lipases could be considered one of the most versatile, they alone can hardly reach the ideal goal of a predominant enzyme synthesis of an API, especially if they involve redox steps; thus, it is necessary to explore the development of enzyme cascades which include the aspects previously listed [236,237,238,239]. Indeed, the implementation of enzyme cascades in the synthesis of APIs with possibilities of industrial exploitation were recently reviewed [240]. Standing out in these was the synthesis of Molnupiravir for the treatment of COVID-19, where the immobilized CAL B lipase (Novozym^®^ 435) participates. This is a paradigmatic example of the way forward in the implementation of lipases in greener processes strongly required in the pharmaceutical industry.

## Figures and Tables

**Table 1:** Some cases of enantioselectivity: explanation, type of reaction and details of the reaction studied.

Enantioselectivity Origins	Lipase	Reaction Type/Conditions	Substrate	Product-Favored	Activity	*ee*%/(*E*)	Reference
Electronic effects: the size and polarity of the stereoselectivity pocket favor interactions with heterocyclic rings.	CAL B W104T	Hydrolysis/Phosphate buffered-saline or PBS (50 mM, pH = 7.5) at 37 °C.	*rac* phenyl(pyridin-4-yl) methyl acetate Related API: (S)-Bepotastine	(S)	No reported (N.R.)	98 (*E* > 200)	[121]
Complementary structure of the acyl site, hydrophobic pocket or oxyanion hole (KRIE): selectivity towards derivatives of chiral acids is increased by replacing large residues with small ones.	CAL B and mutants	Hydrolysis/PBS (50 mM, pH = 7.5) and 1% Triton X-100) at 30 °C.	*rac* p-nitrophenyl 2-phenylpropanoate Related API: Ipatropium	(S)	N.R.	(*E* = 1.2–72)	[120]
H-bonds: the (S) enantiomer is properly oriented to form H-bonds between N(ε) of His and its O alkoxy.	CRL	Esterification/1 volume of cyclohexane with 0.15 M acid and 1 volume 0.11 M decanol aqueous saline solution (a_w_ = 0.16) at RT.	*rac* 2- methyldecanoic acid Related API: Polymyxins	(S)	1.52 µmol.g^−1^·min^−1^	97% (*E* = 90)	[122]
H-bonds as a function of dihedral angle C carbonyl-O alkoxy- Cα-C_β_ alkoxy: the shorter the acyl residue (C2), the dihedral will be closer to 60° which will facilitate the building of an H-bond between O beta as acceptor and the -OH of Ty29 as donor.	*P.cepacia* lipase (PCL)	Hydrolysis/PBS pH 7.0 (100 mM and 10% isopropanol *v/v*) at 25 °C, 200 rpm.	*rac* acyl esters of 2-phenoxypropan-1-ol (acyl n = C2 to C12) Related API: Metoprolol related β-blockers	(S)	0.011 (C12)–0.52 (C2) µmol·min^−1^	*E* values 1,1 (C12) to 33 (C2).	[122]
Steric hindrance or Kazlauskas rule	CAL B	Transesterification/in *t*-BuOMe at 30 °C, 200 rpm with 0.05 mmol substrate and 0.15 mmol vinyl butanoate.	*rac*-1-phenyl-1-ethanol Related API: Norephedrine	(R)	28% conversion in 12 h	>99 (*E* > 200)	[123]
Dynamics of the catalytic cavity “contradicts” the Kazlauskas rule: a rotation of leu278 was presented to give more space to the substrate with the bulky substituent; additional H-bond in the case of the (S) with the oxyanion hole.	CAL B	Transesterification/in *t*-BuOMe at 30 °C, 200 rpm with 0.05 mmol substrate and 0.15 mmol vinyl butanoate.	*rac*-2-amino-1-phenylethanol N-Boc-aminomethylene Related API: Denopamine	(S)	10% conversion in 48 h.	>99 (*E* > 200)	[123]
Mirror-image-packing of the substrate: favored when a stereocenter of the substrate has an H as substituent, explaining a packing of the less favored enantiomer different from that assumed by the Kazlauscas rule (Figure 5).	*Burkholderia cepacia* lipase (BCL)	Hydrolysis/PBS pH 7.0 in 30% n-propanol with 4.5 mmol substrate.	*rac* 2-methyl-3-phenyl-1-propyl heptanoate Related API: Squalestatin	(S)	k_cat_ = 0.4 min^−1^	98 (*E* > 190)	[119]
Substrate structure/presence of inhibitor: by acquiring the hair-pin conformation, the (R)-enantiomer binds to N(ε) of His through its O alkoxy induced by the presence of coordinated heptanol as inhibitor.	CRL	Esterification/1 volume of cyclohexane with 0.15 M acid and 1 volume 0.11 M decanol aqueous saline solution (a_w_ = 0.16) at RT in presence of 900 mM heptanol.	*rac* 2- methyldecanoic acid Related API: Polymyxins	Relative increase (but not absolute) towards the (R) enantiomer.	N.R.	*E* = 37	[122]
Solvation effects/Kazlauskas rule: the reaction medium affects the interaction of the racemic substrate with the solvent, its peers and the enzyme, with the latter, enzyme-substrate diastereomeric complexes with different k_m_ values are formed. The one with the largest km will be the one most affected by the permittivity of the medium depending on the solvent.	CAL B	Transesterification/alcohol 2 mmol and vinyl acetate 8 mmol in toluene−acetonitrile (variable proportion to change polarity, ε) at 30 °C.	*rac* 1-(naphth-2-yl) ethanol or *rac* 2-methoxy-2-phenylethanol Related API: Nifedipine	(R) (R)	100 mol·h^−1^·mg_enz_^−1^ 220 mol·h^−1^*·*mg_enz_^−1^	*E* values from 30–120 (*E* 120 with ε = 12) *E* values from 60–200 (*E* 60 with ε = 14)	[124]
Kazlauskas rule/Higher structural flexibility: in the lid and the entrance to the catalytic cavity (helix α-5) induced by the reaction medium (solvent expanded by CO_2_), it allows better accommodation of bulky substrates, accelerating the reaction.	CAL B	Transesterification/alcohol 0.1 mmol and vinyl acetate 0.2 mmol in 10 mL hexane or CO_2_-hexane (5%, 6 MPa) at 50 °C, 30 min.	*rac* ortho-substituted 1-phenylethanols Related API: Crizotinib	(R)	Conversion rates increased up to 2.5 times in presence of CO_2_-hexane.	>99	[125]

*ee* (%) is the enantiomeric excess percentage; *E* is the enantiomeric ratio.

**Table 2:** Immobilized lipases on hydrophobic interaction and ion exchange supports to obtain APIs.

Derivative	Support Used	Type of Reaction/Conditions	Substrate	Product	Activity (µmol·min^−1^)	*ee*(%)/(*E*)	Uses Related to API
BTL2 immobilized by hydrophobic interaction [149]	Octyl-agarose	Hydrolysis/Acetate buffered-saline or ABS (25 mM, pH = 5.0) at 25 °C.	*rac*-2-O-Butyryl-2-phenylacetic acid	(*R*)-O-Butyryl-2-phenylacetic acid	0.25	>99/*E* > 100	Precursor of mandelic acid derivatives APIs: pemoline ([150], Scheme 7), homatropine, cyclandelate (Scheme 8), cephalosporin, antineoplastics, antiobesity and anti-thrombotic agents [150,151,152]
CAL-B immobilized by hydrophobic interaction [147]	Novozym 435 Octyl-agarose Octadecyl-Sepabeads Butyl-agarose	Hydrolysis/Acetate buffered-saline or ABS (25 mM, pH = 5.0) at 25 °C.	*rac*-2-O-butyryl-2-phenylacetic acid	(S)-2-hydroxy-2-phenylacetic acid (R)-2-hydroxy-2-phenylacetic acid (R)-2-hydroxy-2-phenylacetic acid (R)-2-hydroxy-2-phenylacetic acid	0.75 0.19 0.35 0.16	>99/*E* > 100 95/*E* = 49 90/*E* = 23 72/*E* = 7.5	Precursor of mandelic acid derivatives APIs: pemoline ([150], Scheme 7), homatropine, cyclandelate (Scheme 8), cephalosporin, antineoplastics, antiobesity and anti-thrombotic agents [150,151,152].
TLL immobilized by hydrophobic interaction [154]	octyl-Sepharose^®^	Hydrolysis/Acetate buffered-saline or ABS (25 mM, pH = 5.0) at 25 °C.	β -D-Galactose pentaacetate	C–6 deprotected β -D-Galactose tetraacetylated 99%	N.R.	N/A	Lacto-N-neotetraose in turn precursor of the tumor-associated carbohydrate antigen T and the antitumoral drug [154].
CRL immobilized by ion exchange [165]	Amberjet^®^1200-H (Cation exchanger) Amberjet^®^4200-Cl (Anion ex-changer)	Transesterification/*Rac*-Menthol with vinyl acetate 1:2 in anhydrous solvent at 30 °C.	*rac*-Menthol	(*R*)-(-)- Menthyl acetate	N.R.	>99/ *E* = 208 >99/ *E* = 213	Chiral additive in the resolution of enantiomers of the antiarrhythmic drug encainide and its major metabolites [167].
CRL immobilized by ion exchange [166]	Sephadex^®^ G-25 Sephadex^®^ G-50	Transesterification/*Rac*-Menthol with valeric acetate 1:2 in anhydrous solvent at 30 °C.	*rac*-Menthol	(*R*)-(-)- Menthyl acetate	N.R.	92/ *E* 70.7 90%/ *E* = 33.1	Chiral additive in the resolution of enantiomers of the antiarrhythmic drug encainide and its major metabolites [167].
CAL-B immobilized by ion exchange [168]	PEI-Agarose at 4 °C PEI-Agarose at 25 °C PEI-Agarose at 37 °C	Hydrolysis/Acetate buffered-saline or ABS (25 mM, pH = 5.0) at 25 °C	*rac*- mandelic acid methyl ester	(*R*)-2-hydroxy-2-phenylacetic acid	0.21 0.21 0.39	*E* = 25 *E* = 16 *E* = 10.5	Precursor of mandelic acid derivatives APIs: pemoline ([150], Scheme 7), homatropine, cyclandelate (Scheme 8), cephalosporin, antineoplastics, antiobesity and anti-thrombotic agents [150,151,152].
CRL immobilized by ion exchange [169]	DEAE-glycidyl methacrylate (GMA)- ethylene dimethacrylate (EDMA) activated	Transesterifica-tion/Rac-Menthol with propionic anhydride 1:1 in anhydrous solvent at 30 °C.	*rac*-Menthol	(*R*)-(-)- Menthyl acetate	N.R.	>90	Chiral additive in the resolution of enantiomers of the antiarrhythmic drug encainide and its major metabolites [167].

*ee* (%) is the enantiomeric excess percentage; *E* is the enantiomeric ratio.

**Table 3:** Intensity of enzyme-support union in BTL2 covalent derivatives [183,184].

Derivative (Agarose Matrix)	Type of Covalent Union/Immobilization Conditions	Thermal Stability at 70 °C t_½_ (min)	Resolution of *ee*-2-O-butyryl-2-phenylacetic Acid (“a”). *ee*_(R)_ (%)/Activity (µmol·min^−^^1^ mg_lipase_^−1^)	Desymmetrization of Phenyl Glutaric Acid Methyl Diester (“b”) *ee*_(S)_ (%)/Activity (µmol·min^−^^1^ mg_lipase_^−1^)
CNBr (Cyanate ester)	Monopoint (via N-terminal)/pH 7 in 50 mM PBS at 4 °C.	26	24/(0.71)	94/(0.51)
Disulfide (-S-S-)	Monopoint through the introduced cysteine (Q40C C65S, C296S BTL2)/at pH 7 in PBS 50 mM at 4 °C.	21	45/(0.56)	91/(0.47)
Glyoxyl (aldehyde)	Non oriented multipoint through Lys/N-terminal residues/At pH 10 in sodium carbonate 100 mM (25 °C).	602	80/(0.68)	>99/(0.44)
Glyoxyl + disulfide	Oriented through cysteine (Q40C) plus soft multipoint union through adjacent native ε-amino groups./at pH 7 in PBS 50 mM and then pH increased to 10 in sodium carbonate 100 mM (25 °C).	510	>99/(0.59)	96/(0.48)
Glyoxyl + disulfide	Oriented through cysteine (Q40C) plus intense multipoint union through adjacent native and other amino groups introduced chemically/at pH 7 in PBS 50 mM and then pH increased to 10 in sodium carbonate 100 mM (25 °C).	1010	>99/(0.29)	81.8/(0.86)
Glyoxyl + disulfide	Oriented through cysteine (Q40C) plus intense multipoint union through adjacent native and other amino groups introduced chemically and genetically (Arg→Lys in positions 374, 379, 48, 35, 22 and 5)/at pH 7 in PBS 50 mM and then pH increased to 10 in sodium carbonate 100 mM (25 °C).	380	>99/(0.09)	>99/(0.11)

Hydrolysis conditions: catalyst (0.3 g) or soluble enzyme (1 mg/mL) (0.4 mL) in 2 mL solutionof 1 mM (±)-“a” in sodium acetate (25 mM) at pH 7 and 25 °C or immobilized catalyst (0.42 g) or soluble enzyme (0.6 mL) in 6 mLsolution of 2 mM of “b” in sodium acetate (25 mM) at pH 7.0 and 25 °C. The hydrolysis degrees were calculated between 15 and 20%, where the reaction kinetic is in first order [183,184]. *ee* (%) is the enantiomeric excess percentage.

**Table 4:** Immobilized lipases to obtain APIs [12].

Derivative/Immobilization Conditions	Commercial Name	Type of Reaction/Conditions	Substrate	Product/Yield, *ee%*/(E)	Uses Related to API
CAL B immobilized by hydrophobic interaction on Lewatit^®^ VP OC 1600/conditions non specified	Novozym^®^ 435	Hydrolysis Amidation/PBS buffer pH 6.5–7.5 at 30–35 °C (24–48 h).	Methyl *rac*-2-bromobutyrate	(R)-methyl 2-bromobutanoate/No reported (N.R.)	Production of anti-epileptic drug levetiracetam (Elepsia^®^ o Keppra^®^) [12]
CAL B immobilized/type or conditions non specified	Chirazyme L2^TM^	Hydrolysis resolution/Buffer pH 7.0/MTBE(5:1, *v/v*) at 20 °C.	*rac*-1-({[(1,1,1,3,3,3-hexafluoropropan-2-yl)oxy]carbonyl}oxy)ethyl 2-methylpropanoate	(*R*)-1-((((1,1,1,3,3,3-hexafluoropropan-2-yl)oxy)carbonyl)oxy)ethyl isobutyrate and (S)-1,1,1,3,3,3-hexafluoropropan-2-yl (1-hydroxyethyl) carbonate/Yield 44%, *ee* 99%.	Production of a prodrug type carbamate with inhibitory activity of TAFIa (thrombin-activatable fibrinolysis inhibitor) for the treatment of thromboembolic disorders [12]
TLL immobilized by cationic exchange in silica/conditions non specified	Lipozyme^®^ TL IM Lipozyme^®^ RM IM Lipase PS IM	Enatioselective hydrolysis	Precursor esters of valsartan	Valsartan/Yield of 90–95%ee(S) = 99.5	Enatioselective hydrolysis of esters of valsartan (Diovan^®^, a drug that modulates angiotensin-renin system to treat hypertension [201,202]
CAL B immobilized by hydrophobic interaction on Lewatit^®^ VP OC 1600/conditions non specified	Novozym^®^ 435	Chemoselective hydrolysis of esters/PBS pH 8.0 and organic solvent (1:1 *v/v*)	N-Boc-(1R,3S,4S)-2-azabicyclo[2.2.1]heptane-3-carboxylate	Carboxylic derivative/Yield > 70%, diastereomeric excess values of 99.7%	Production of a building block required for the preparation of ledispavir (Harvoni, Gilead, along with sofosbuvir for the treatment of hepatitis C) [12]
CAL B immobilized by hydrophobic interaction on Lewatit^®^ VP OC 1600/conditions non specified	Novozyme^®^ 435	Stereoselective acylation/with isopropanoic anhydride in a mixture of NaHCO_3_/toluene with vinyl acetate at 15 °C during 24 h.	prochiral 2-(2-(2,4-difluorophenyl)allyl)propane-1,3-diol	(S)-4-(2,4-difluorophenyl)-2-(hydroxymethyl)pent-4-en-1-yl isobutyrate/Yield 75%	Production of a precursor of the antifungal posaconazole (Posanol^®^), Noxafil^®^) [203]
CAL B immobilized by hydrophobic interaction on Lewatit^®^ VP OC 1600/conditions non specified	Novozyme^®^ 435	Transesterification/ tolunene with perchlorophenyl acetate at 50–60 °C.	*rac*-3-cyano-2-hydroxy-N,N,N-trimethylpropan-1-aminium	(R)-2-acetoxy-3-cyano-N,N,N-trimethylpropan-1-aminium	Obtaining acetate (2R) as a precursor of L-carnitine, key in lipid metabolism and sold as a supplement [203]
CAL B immobilized by hydrophobic interaction on Lewatit^®^ VP OC 1600/conditions non specified	Novozyme^®^ 435	Hydrolysis of a uracil nucleoside/methoxypropanol/water at 60 °C, 48 h.	2-methyl-2-fluoro-deoxyribose peracetylate	deacetylated derivative selectively in position C-5′/yield > 99%	obtaining a precursor of Sofosbuvir, a drug used in the treatment of chronic hepatitis [203]
CAL B immobilized by hydrophobic interaction on Lewatit^®^ VP OC 1600/conditions non specified	Novozym^®^435	Dynamic nantioselective acetylation/Toluene with (R)-O-acetyl mandelic acid, H_2_/nickel catalyst (KT-02), 1 MPa H_2_, at 70 °C, 24 h.	*rac*-2-aminotetraline	(*R*)-2-aminotetraline/ee > 99%	Obtention of a pharmacophore based stimulant drugs with action on dopamine receptor D3 [204]
CAL B immobilized by hydrophobic interaction/conditions non specified	Novozym^®^435	Monoacetylation in C-6/THF, 40 °C, 60 h.	β-D-glucosides polydatin and geniposide	Acylated derivatives of β-D-glucosides polydatin and geniposide/Yield 78%	Acylated derivatives of β-D-glucosides polydatin (anti-inflammatory, anti-oxidant and in anti-angiogenesis chemotherapy) and geniposide (used in the prevention and therapy of hepatic injury-HI) [12]

*ee* (%) is the enantiomeric excess percentage; E is the enantiomeric ratio.

## Data Availability

Not applicable.
